# Evolutionary Characteristics of the Rice SnRK2 Family and ABI5 Interaction Analysis Reveal Their Key Regulatory Role in Osmotic Stress Signaling

**DOI:** 10.3390/ijms27156788

**Published:** 2026-07-29

**Authors:** Nachuan Zhang, Xinye Xu, Yixin Zhang, Jiangyue Niu, Xinlian Liu, Lijia Li, Jiaqi Hou

**Affiliations:** State Key Laboratory of Hybrid Rice, College of Life Sciences, Wuhan University, Wuhan 430072, China; nachuanzhang@whu.edu.cn (N.Z.); xinlianliu@whu.edu.cn (X.L.);

**Keywords:** SnRK2, ABI5, ABA, rice, protein interaction, phylogenetic tree

## Abstract

The ABA signaling pathway plays a central role in the regulation of plant responses to stress, with ABI5 serving as an important transcription factor within this pathway and exerting a key regulatory function. However, upstream kinases that regulate ABI5 have not been fully elucidated, especially the specific relationship between ABI5 and SnRK2 members in rice. Rice contains ten OsSAPK family members, which are classified into three conserved phylogenetic groups, including Group I (OsSAPK1/2/3), Group II (OsSAPK4/5/6/7), and Group III (OsSAPK8/9/10). This study aims to identify potential OsABI5-interacting kinases involved in ABA-mediated salt stress responses in rice. Phylogenetic analysis confirmed that the ten OsSAPKs were distributed into three conserved groups. Phenotypic analysis showed that ABA pretreatment alleviated salt-induced growth inhibition in rice seedlings, while RT-qPCR analysis revealed increased transcript levels of several OsSAPKs following ABA pretreatment under salt stress. Promoter analysis identified multiple hormone-responsive cis-elements in *OsSAPK* promoters, consistent with their transcriptional responses to ABA treatment. Integrated protein–protein interaction analyses revealed that OsSAPK1/3/4/9 physically bind to OsABI5, as demonstrated by complementary in vitro and in vivo assays. These findings identify four candidate OsABI5-interacting kinases and reveal potential SnRK2–ABI5 regulatory mechanisms in ABA-mediated salt stress responses.

## 1. Introduction

Abscisic acid (ABA), a crucial plant hormone, functions as a central regulator in the adaptation of plants to various abiotic stresses [1]. Upon exposure to adverse environmental conditions, plants rapidly enhance ABA biosynthesis and accumulation, thereby activating a conserved signaling cascade that promotes stress tolerance through extensive transcriptional reprogramming [2]. The classical ABA signaling pathway follows a well-defined perception–transduction–response regulatory chain. In the absence of ABA, PP2Cs maintain SnRK2s in an inactive state through dephosphorylation. Following ABA recognition by PYR/PYL/RCAR receptors, PP2C inhibition allows Sucrose Non-Fermenting 1-Related Protein Kinase 2 (SnRK2) activation, triggering downstream ABA-responsive signaling events. Subsequently, the self-phosphorylated-activated SnRK2 phosphorylates a series of downstream target elements, including numerous members of the bZIP family, ultimately contributing to the activation of downstream transcriptional networks involved in plant adaptation to adverse environmental conditions [3,4]. The phosphorylated bZIP transcription factor coordinates the adaptive stress response in plants by activating the transcription of stress-related genes containing ABA response elements (ABRE) [5,6].

In plants, the SNF1-associated protein kinase (SnRK) family represents an important class of serine/threonine kinases involved in diverse regulatory processes, which is mainly involved in stress signaling and metabolic regulation. Based on phylogenetic relationships and functional characteristics, members of the SnRK family fall into three subfamilies. SnRK1 primarily functions as an energy sensor, SnRK2 is involved in the salt stress response (covering both ABA-dependent and non-dependent pathways), while SnRK3 (CIPK/PKS) regulates ion homeostasis mainly in response to Ca^2+^ signals [4,7]. This study focused on the SnRK2 subfamily. Members of this family have been characterized in a range of plant species, including major cereal crops and cash crops such as soybeans, rice, tomatoes, wheat, and maize [4,8,9,10,11]. Studies in model plants, such as *Arabidopsis thaliana* and *Physcomitrella patens*, have revealed that SnRK2 kinases represent an evolutionarily conserved family in terrestrial plants [12,13]. As key regulators of ABA signaling, SnRK2s coordinate multiple stress-responsive processes, particularly salt stress adaptation, through functional diversification among family members [14,15]. In *A. thaliana*, SnRK2.2/2.3/2.6 regulate ABA-induced stomatal closure, while SnRK2.4 and SnRK2.10 contribute to root development and salt tolerance. Our research focused on rice (*Oryza sativa* L.), and because most of its members can be driven by ABA and abiotic stress, the SnRK2 family encoded by rice is often referred to as stress-activated protein kinases [16]. The stress-activated protein kinases (SAPKs) family consists of ten members (SAPK1 to 10). Phylogenetic analysis divides the family into three subpopulations, with subpopulation III being phylogenetically closest to the core ABA response kinase in *A. thaliana* and therefore presumed to play a central role in ABA signaling, including *OsSAPK8/9/10* [16,17]. Several OsSAPK members have been functionally characterized. OsSAPK6 has been implicated in drought tolerance, whereas OsSAPK8/10 participate in ABA signaling and stomatal regulation under abiotic stress conditions [18,19,20].

Among the ABA-responsive transcription factors, ABA insensitive factor 5 (ABI5) occupies a particularly critical position. Initially discovered from *A. thaliana ABI5* mutants [21], ABI5 exhibits ABA-responsive expression patterns and accumulates during key developmental stages, including seed maturation, dormancy, germination, and seedling stress adaptation [22]. Phosphorylated ABI5 can form homodimers or heterodimers with other bZIP transcription factors, which specifically recognize ABREs in downstream target gene promoters, thereby activating the transcription of late embryogenesis abundant (LEA) proteins, early methionine marker proteins, and various antioxidant enzymes [23,24,25]. This activity is crucial for inhibiting seed germination, blocking seedling growth, and establishing basal stress resistance [26,27]. Therefore, ABI5 is regarded as a core terminal hub that integrates upstream ABA signaling with downstream stress-response gene transcription networks.

Although several OsSAPKs have been individually implicated in ABA signaling and abiotic stress responses, previous investigations have predominantly examined the functions and characteristics of individual family members [18,19,20]. However, comparative analyses encompassing the entire OsSAPK family remain limited. In particular, the rice genome encodes ten OsSAPK family members, yet the identities of those capable of physically associating with OsABI5 and the potential relationships between their evolutionary characteristics, promoter composition, and expression patterns have not been systematically investigated. A comprehensive comparison of these characteristics may help prioritize candidate OsABI5-interacting kinases for subsequent functional studies. Therefore, it is currently unclear which members are most likely to bind to the downstream transcription factor OsABI5. Additionally, whether the evolutionary relationship promoter characteristics and transcriptional responses of different OsSAPKs are consistent with their potential OsABI5 interaction capabilities has not been fully studied. This study fills this gap by focusing on the physical interaction between OsSAPK members and ABI5, and identifies a specific kinase subgroup responsible for regulating ABI5 under salt stress.

To elucidate the characteristics and potential functions of OsSAPKs in rice, we employed a combination of evolutionary, transcriptional, and protein interaction analyses. We first analyzed the phylogenetic relationships, conserved protein motifs, and sequence characteristics of OsSAPKs to evaluate their evolutionary conservation. We then assessed the effects of ABA pretreatment on rice seedling growth under salt stress and examined the transcriptional responses of OsSAPKs by RT-qPCR. Promoter cis-element analysis was further performed to characterize potential regulatory features within *OsSAPK* promoter regions [28,29]. Finally, by utilizing a combination of various protein interaction detection methods, including yeast two-hybrid analysis, pull-down assays, and bimolecular fluorescence complementation (BiFC) assays, the physical interaction between OsSAPK and OsABI5 was characterized. The results identify candidate OsABI5-interacting kinases and facilitate further functional studies of SnRK2–ABI5 associations in ABA-mediated salt stress responses in rice.

## 2. Results

### 2.1. Classification of the SnRK2 Family in Plants

**Figure 1:**
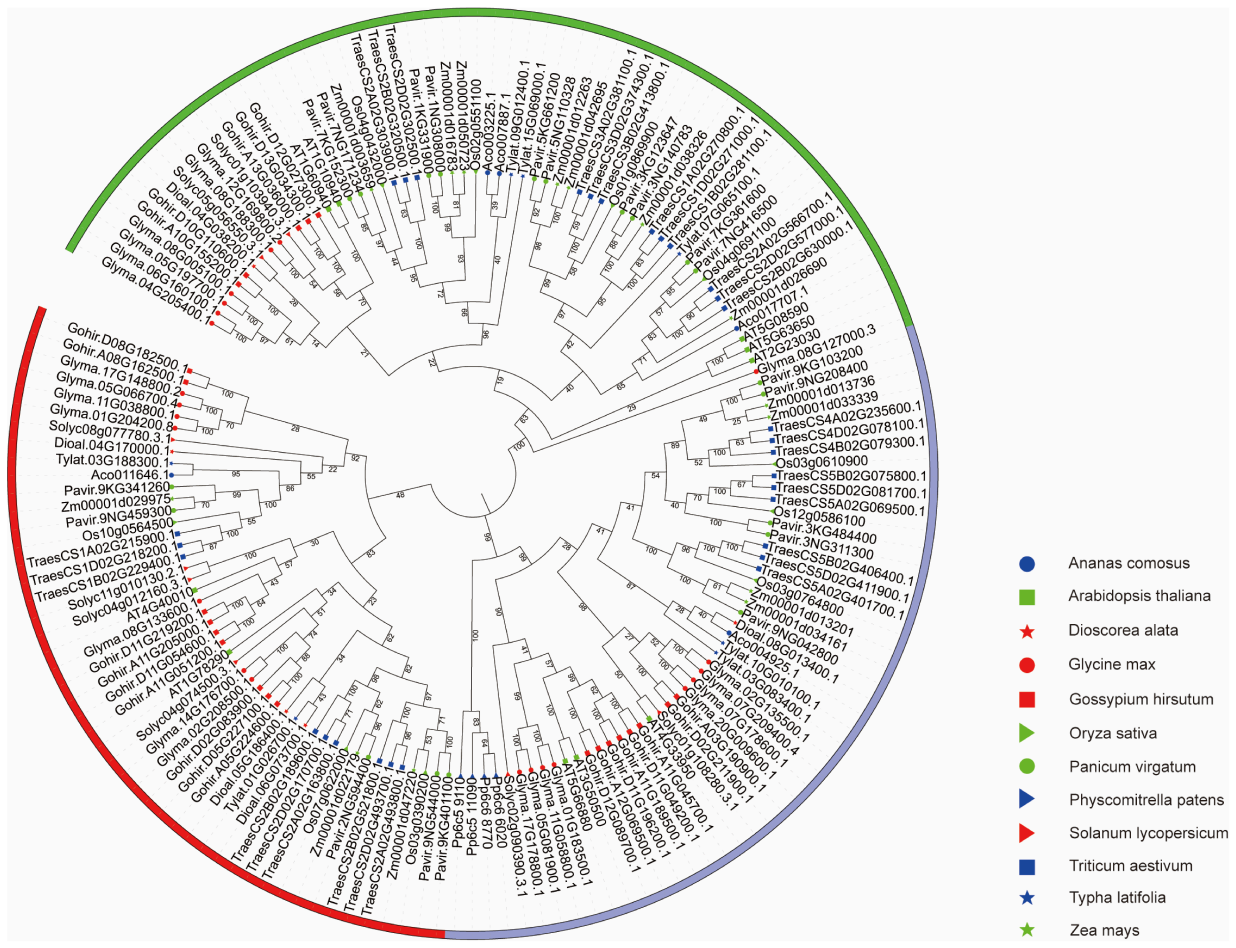
Phylogenetic analysis of SnRK2 in plants. SnRK2 are divided into three subfamilies: red section for Group I, green section for Group II, and blue section for Group III. SnRK2 members of different species are marked by geometric figures of different colors. Data from the Phytozome database.

**Figure 2:**
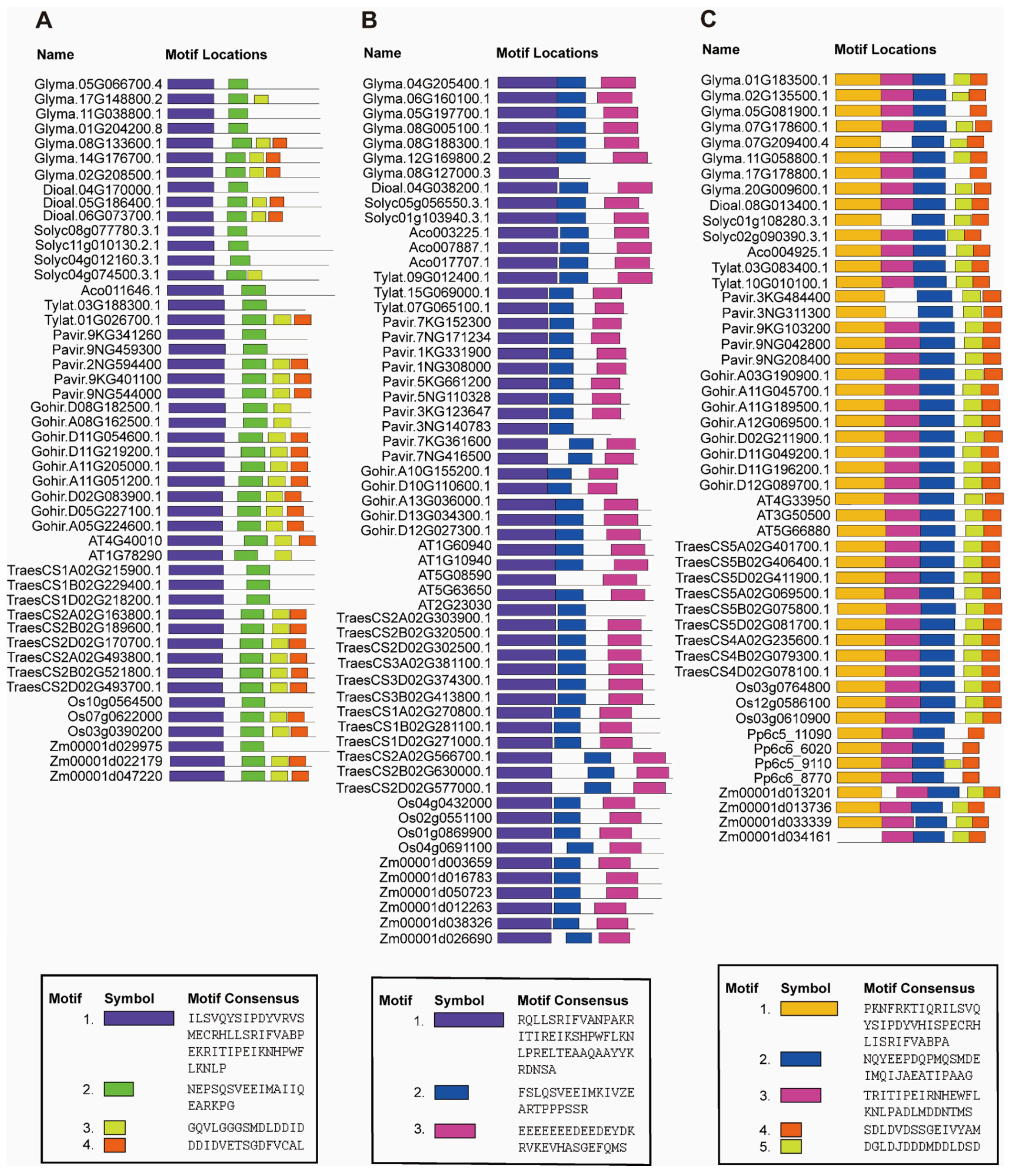
Motif analysis of the amino acid C-terminal sequences. This study identified plenty of motifs in plant SnRK2, which were classified into three phylogenetic groups (shown in (**A**–**C**)). Each color band represents a motif, and the black underline (blank) represents the amino acid sequence between the motifs. The symbol and sequence of the motifs are also listed.

To investigate the evolutionary relationships within the SnRK2 family, we characterized 157 SnRK2 members’ amino acid sequences from 12 representative plant models and performed a phylogenetic analysis (Figure 1). The genomic sequences were acquired from the publicly accessible Phytozome database, and a summary of the BLASTP results used to identify SnRK2 homologs in the selected plant species is provided in Table S1. The results show the classification of the SnRK2 family into three distinct subgroups, Group I to III (Figure 1). The MEME analysis was subsequently performed to characterize conserved motifs from the C-terminal sequences of amino acids of SnRK2 members in these plants (Figure 2). These members showed a subgroup distribution of four, three, and five proteins in Groups I, II, and III, respectively. In addition, we performed multiple sequence alignment using MEGA12 software to further confirm the conservatism of SnRK2 in the group. In this study, Ser/Thr protein kinase activity sites and ATP-connecting sites were identified in all plant SnRK2 members (Figure S1). These active sites may be characterized by the traditional functional effects of the SnRK2 family, which are highly conserved. Members of different subgroups are proposed to exhibit varying degrees of dependence on the phytohormone abscisic acid for their activation, thereby playing divergent roles within the ABA signaling network [16,30]. Consistent with this functional classification, genetic and biochemical studies in *A. thaliana* have identified SnRK2.2, SnRK2.3, and OST1 (SnRK2.6) as core components that are highly ABA-dependent [6,30]. Similarly in *Zea mays*, SnRK2.8 (Zm00001d013201), SnRK2.9 (Zm00001d013736), and SnRK2.10 (Zm00001d034161) have been characterized as ABA-responsive kinases [8]. These previously characterized ABA-responsive SnRK2 members are all classified within Group III according to our phylogenetic analysis. In *Oryza sativa*, SnRK2 proteins are commonly referred to as stress-activated protein kinases (SAPKs). Three members cluster within Group III and have been experimentally confirmed to be activated by ABA, including OsSAPK8 (Os03g0764800), OsSAPK9 (Os12g0586100), and OsSAPK10 (Os03g0610900) [16]. Based on this phylogenetic framework, subsequent analyses were conducted to compare the expression characteristics and OsABI5 interaction profiles of all OsSAPK family members.

### 2.2. Phenotypic Assays and Expression Profiling of OsSAPK Members Under Salt Stress

**Figure 3:**
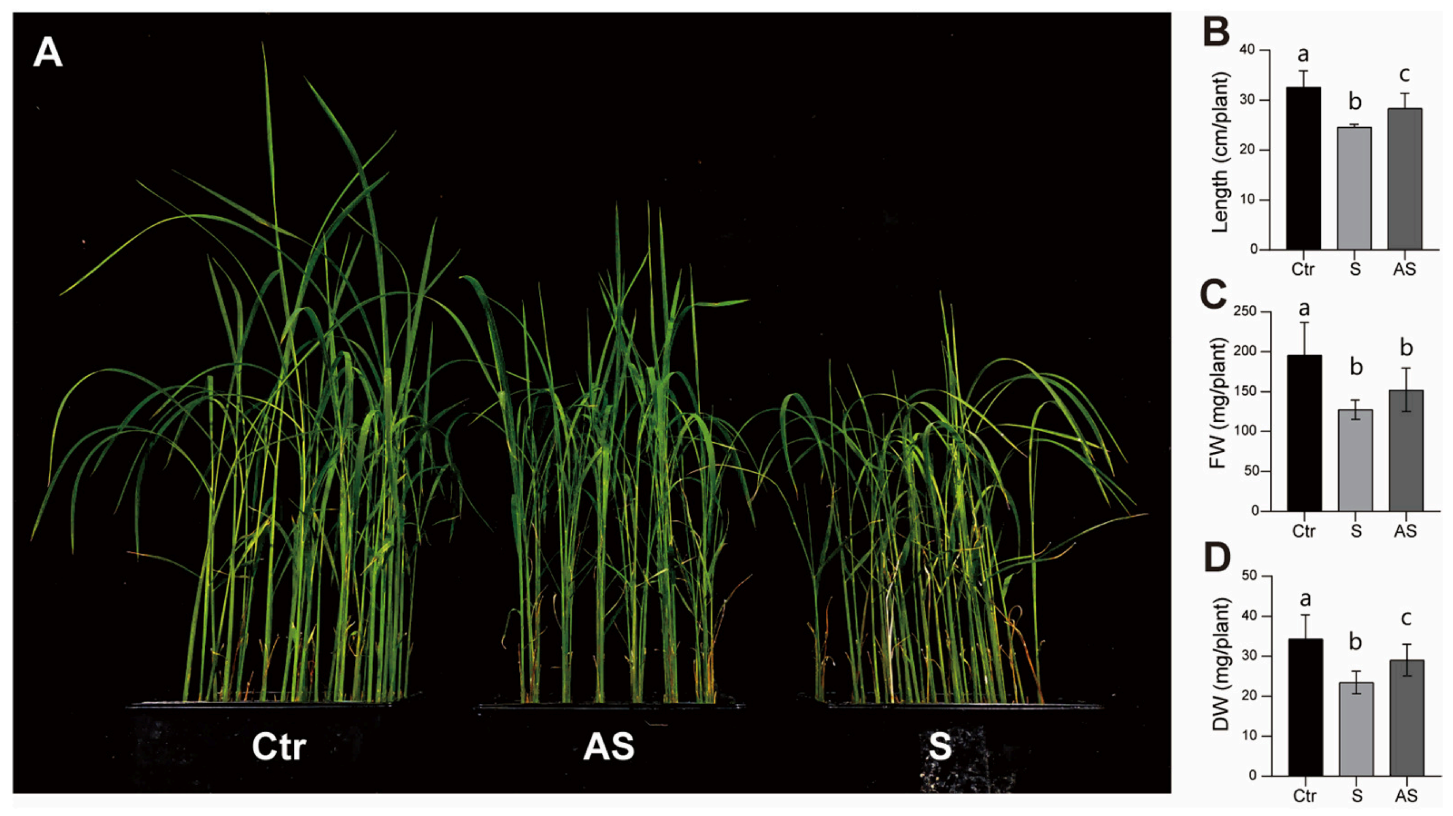
Phenotypic responses of rice seedlings under salt stress and ABA pretreatment. (**A**) Rice seedlings grown for 14 days (NIP) under standard nutrient solution. Untreated group (Ctr), ABA pretreated for salt stress group (AS), and salt stress group (S) are shown from left to right. (**B**–**D**) Statistics regarding the length, fresh weight (FW), and dry weight (DW) of the above-ground parts for each group of seedlings. Five biological replicates were collected. Different lowercase letters denote significant differences between treatments based on one-way ANOVA followed by Tukey’s multiple comparison test (*p* < 0.05).

One of the main threats that affects rice growth is salt stress. After 2 days of salt treatment, 14-day-old rice seedlings exhibited obvious growth inhibition compared with the untreated control (Ctr). However, ABA solution pretreatment (1 μM) markedly alleviated injuries caused by adverse stress (AS) (Figure 3A). Furthermore, although ABA did not significantly increase the fresh weight of rice seedlings, the above-ground height and dry matter weight of the ABA pretreated salt stress group were significantly higher than those of the salt stress group (*p* < 0.05). These observations indicate that salt stress reduced seedling growth, whereas ABA pretreatment was associated with partial recovery of above-ground growth under the experimental conditions used (Figure 3B–D). In plant stress signaling pathways, the transcriptional upregulation of key regulatory components often indicates their central functional role in cascade reactions [6].

**Figure 4:**
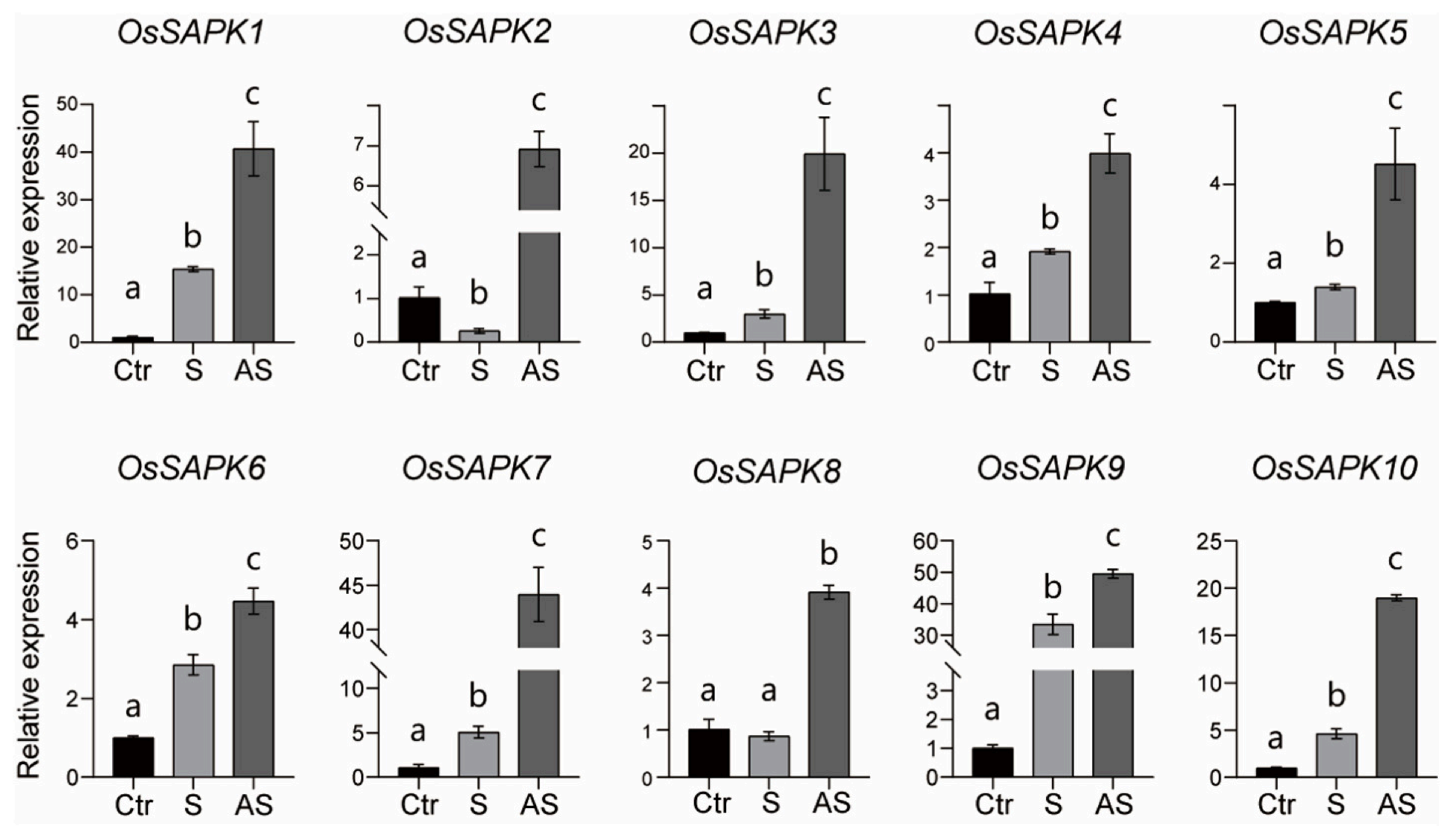
Expression profiling of *OsSAPK* members under salt stress and ABA pretreatment. RT-qPCR analysis of *OsSAPK1* to *10* in Ctr, S, and AS groups. The data were normalized to *ubiquitin* (LOC_Os03g13170) expression, and the relative gene expression was set to 1 for the Ctr. The bar height in the figure shows the average value obtained from three independent biological samples of each group of samples ± SD, averaging their 2^−ΔΔCt^. Different lowercase letters denote significant differences between treatments based on one-way ANOVA followed by Tukey’s multiple comparison test (*p* < 0.05). Primer sequences are listed in Table S3.

To further characterize the molecular responses of each *OsSAPK* family member associated with these treatments, we analyzed their relative transcriptional changes. We compared the expression levels of all ten *OsSAPK* members in rice seedlings using RT-qPCR (Figure 4). The results revealed that after two days of ABA pretreatment (AS), the expression levels of all *OsSAPK* members increased significantly. In addition, all members except OsSAPK2 and OsSAPK8 also showed increased transcript abundance in NaCl-exposed seedlings relative to non-stressed plants [31]. These expression profiles provided the basis for subsequent promoter cis-element analysis.

### 2.3. cis-Element Analysis of OsSAPK Promoters

**Figure 5:**
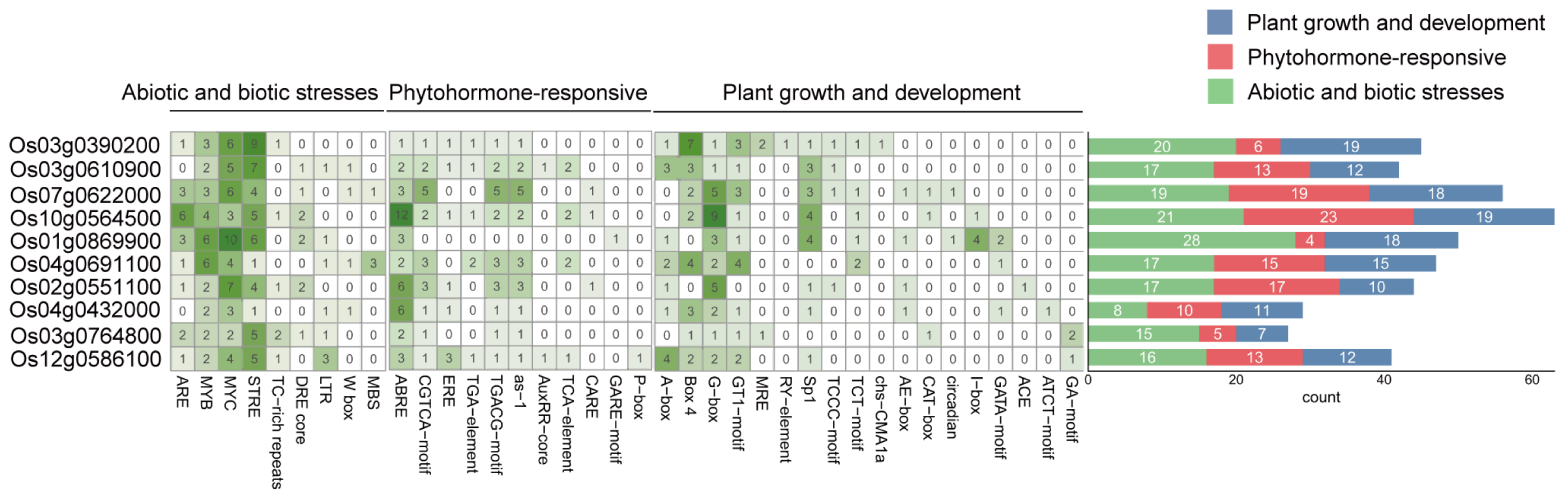
Cis-element analysis of *OsSAPK* promoters in *Oryza sativa*. Heatmap shows the occurrence of predicted cis-acting elements identified in the promoter region of each *OsSAPK* gene. The numbers in the table represent the number of cis-elements that are included and are clearly visualized by color shades. The response elements are displayed at the top of the table. The stacked bar chart summarizes the total number of cis-elements in each functional category for each *OsSAPK* promoter.

Promoter cis-acting elements are important regulatory sequences that are associated with transcriptional responses throughout developmental processes and in response to diverse environmental factors. To further characterize the regulatory features of *OsSAPKs*, potential cis-regulatory elements in the promoter region of the *OsSAPK* gene were further identified using PlantCARE software. The complete list of predicted cis-acting elements is provided in Table S2. By retrieving and analyzing the upstream sequences of *OsSAPK* members, the types and distribution patterns of potential regulatory motifs were identified (Figure 5). According to their annotated functions, these cis-elements were assigned to three main categories based on their predicted functions: abiotic and biotic stress, phytohormone-responsive, and plant growth and development response elements. Multiple cis-regulatory elements that may contribute to stress adaptation and hormonal regulation were detected within upstream regions of several *OsSAPKs*, including STRE, ABRE, MYB, and MYC motifs. Compared with other *OsSAPKs*, *OsSAPK1/3/4/9/10* displayed greater variation in their cis-element profiles. Consistent with the transcriptional responses observed in Figure 4, several of these genes also exhibited increased transcript abundance following ABA pretreatment under salt stress. These observations provide complementary information for prioritizing candidate OsSAPKs for subsequent protein–protein interaction analyses.

### 2.4. Preliminary Screening of OsSAPK Proteins Interacting with OsABI5 by Yeast Two-Hybrid and Planta LCI Assay

**Figure 6:**
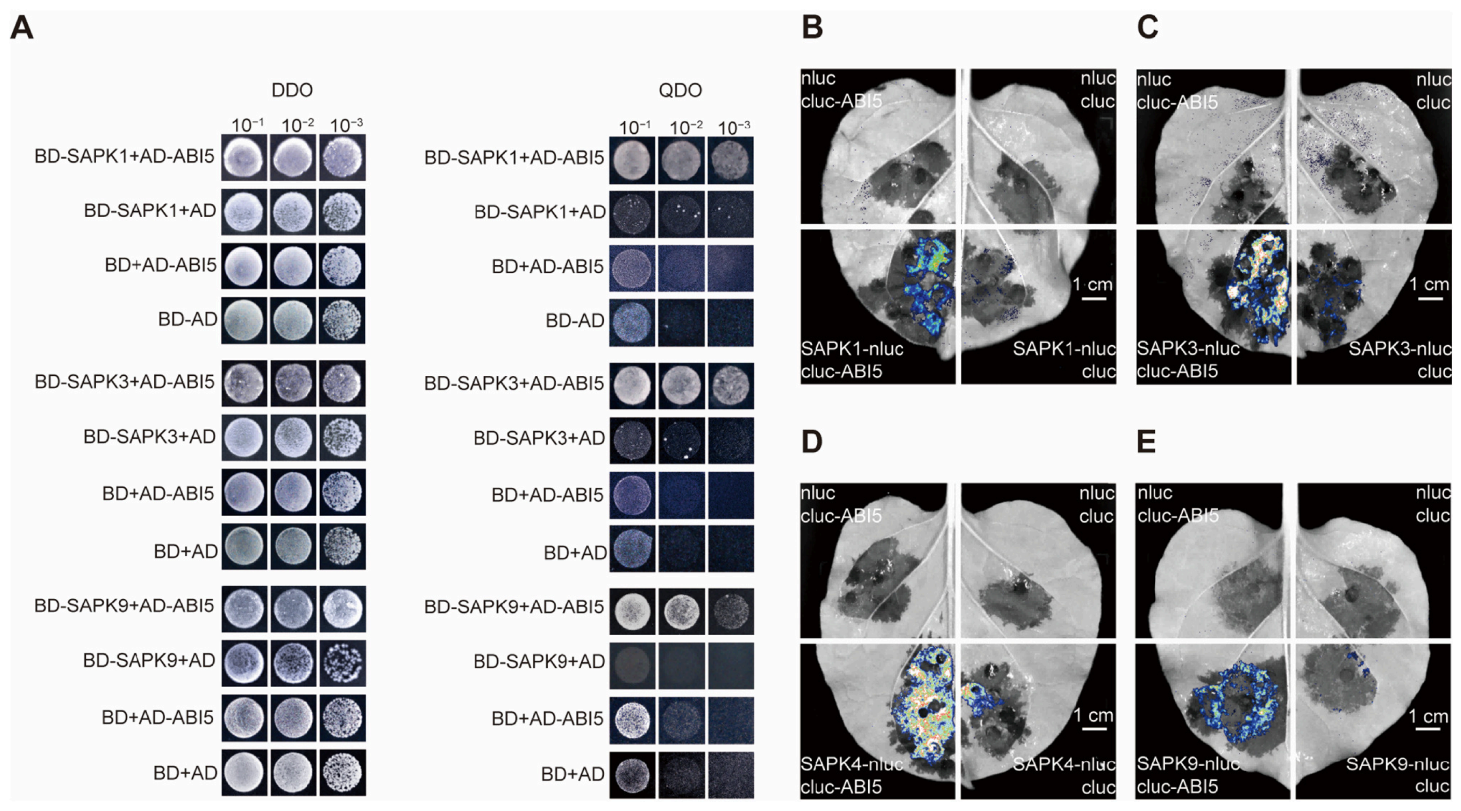
Assays to screen OsABI5-interacting OsSAPK members. (**A**) Y2H assay of interaction. Double drop out medium (DDO): SD/-Leu-Trp; quarter drop out medium (QDO): SD/-Leu-Trp-Ade-His. pGADT7/pGBKT7 empty vector were used as negative control. Each group has three biological replicates. Samples were subjected to a series of sequential ten-fold dilutions for each group. (**B**–**E**) Imaging results of the *N. benthamiana* luciferase complementation experiment. Scale bars represent 1 cm.

In the canonical ABA signaling pathway, SnRK2 protein kinases interact with and phosphorylate downstream bZIP transcription factors such as ABI5 [4,5]. To systematically identify potential interacting proteins within the rice SnRK2 family, we employed yeast two-hybrid and luciferase complementary imaging detection to screen for protein–protein interactions between OsABI5 and ten OsSAPK members [32]. Results of the Y2H assay show that three members showed positive growth on the selected medium among the ten OsSAPKs tested, including OsSAPK1, OsSAPK3, and OsSAPK9, which indicated a potential interaction with OsABI5 in the yeast system (Figure 6A). It is worth noting that OsSAPK1, OsSAPK3, and OsSAPK9 also exhibited marked transcriptional upregulation in the expression analysis (Figure 4). To further evaluate these interactions, a luciferase complementation imaging (LCI) assay was performed in *Nicotiana benthamiana* leaves. A clear luminescent signal was detected in leaves co-expressing cLUC-OsABI5 with each of the four OsSAPK-nLUC fusions (OsSAPK1/3/4/9) 48 h post-infiltration. Negative control combinations showed negligible background, with an empty nLUC vector (Figure 6B–E). Taken together, the Y2H and luciferase complementation assays consistently identified OsSAPK1/3/4/9 as candidate OsABI5-interacting proteins.

### 2.5. Identification of OsABI5-Interacting OsSAPK Members by Pull-Down and Co-Immunoprecipitation Assay

**Figure 7:**
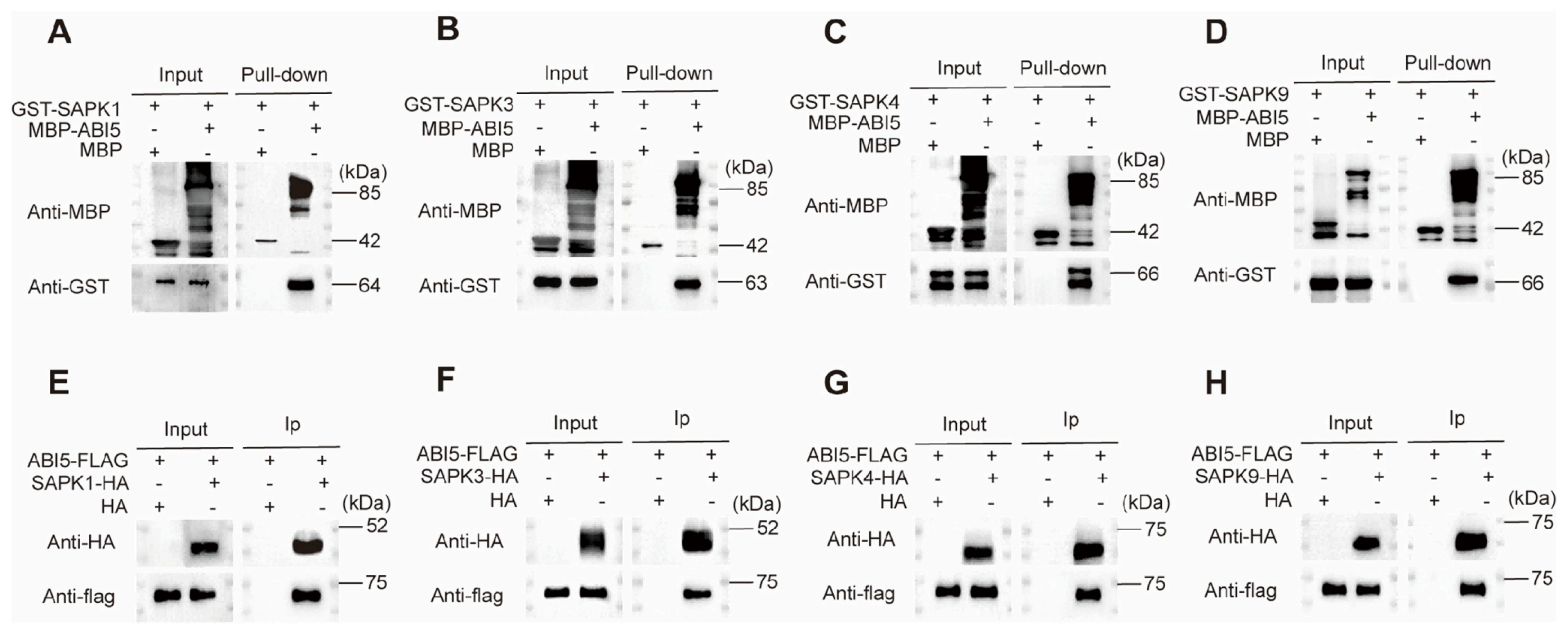
Protein–protein interaction assays to identify OsABI5-interactors. (**A**–**D**) Pull-down assay of GST-OsSAPK1/3/4/9 and MBP-OsABI5 indicates in vitro interaction. (**E**–**H**) In vivo co-immunoprecipitation (Co-IP) assay in a *N. benthamiana* transient expression system.

Based on previous yeast double hybridization (Y2H) and Planta LCI assays, four OsSAPKs (OsSAPK1/3/4/9) were selected for further interaction validation. To further validate these interactions in vitro and in vivo, pull-down assays using purified recombinant proteins and co-immunoprecipitation (Co-IP) assays in a tobacco transient expression system were performed. The results of the pull-down assay indicated that the four candidate members identified in the previous screening were successfully co-precipitated with the OsABI5-MBP bait protein, whereas no obvious interaction signal was detected in the MBP control sample (Figure 7A–D). The corresponding uncropped immunoblot images are provided in Supplementary Figure S2. This independent in vitro validation significantly supports the credibility of the initial interactions screening. To validate the precipitation results from a biochemical perspective, we conducted co-immunoprecipitation assays (Co-IP) in a tobacco transient expression system [33,34]. Results indicated using anti-HA magnetic beads successfully co-precipitated OsABI5-FLAG with OsSAPK1/3/4/9-HA, but not with the HA empty vector control (Figure 7E–H). All constructs had equivalent protein expression levels in the input samples, confirming the specificity of the interaction. These results directly provided biochemical evidence that all four OsSAPK proteins can form complexes with OsABI5 in the plant cell environment.

## 3. Discussion

The plant hormone abscisic acid (ABA) is essential for osmotic pressure responses in plants, and its core signaling pathway has been extensively characterized in *A. thaliana* and other model dicots. The ABA signaling pathway relies on SnRK2-mediated phosphorylation events that influence the transcriptional activity of bZIP factors, including ABI5, thereby activating the transcription of responsive genes. However, direct physical interactions between OsSAPKs and OsABI5 remain largely unexplored in rice. In this study, various methods for analyzing protein–protein interactions were utilized to identify and confirm candidate members of the OsSAPK family that can interact with OsABI5. These findings lay the foundation for further investigations into the role of the SnRK2–ABI5 regulatory module in the response of rice to ABA.

Our phylogenetic analysis of 157 SnRK2 proteins from 12 representative plant species further confirmed the evolutionary conservation of the SnRK2 family and supported the classification of plant SnRK2s into three major subgroups (Figure 1). Within this phylogenetic framework, previous studies in *Arabidopsis thaliana* have established that Group III SnRK2 members function as major components of ABA signaling and directly associate with downstream transcription factors such as ABI5 and ABFs [4,35]. Consistent with this conserved model, several rice Group III SAPKs, particularly OsSAPK8, OsSAPK9, and OsSAPK10, have been reported to participate in ABA-responsive stress regulation [18,19]. Therefore, Group III OsSAPKs have been considered the most likely candidates for functional association with OsABI5 in rice. However, our interaction analyses revealed that OsABI5-associated candidate kinases were not limited to Group III members. In addition to OsSAPK9, which belongs to Group III, OsSAPK1 and OsSAPK3 from Group I and OsSAPK4 from Group II also showed reproducible interactions with OsABI5 in multiple protein–protein interaction assays (Figure 6 and Figure 7). Although Group I and Group II SnRK2 members have traditionally received less attention in ABA-dependent signaling compared with Group III kinases, increasing evidence indicates that these subgroups may participate in diverse stress-related regulatory processes beyond the classical ABA pathway [36,37]. In this context, the interaction patterns observed in our study suggest that the potential association between SnRK2 kinases and OsABI5 may involve a broader range of family members in monocot plants than previously recognized. These results indicate that the ABA signaling regulation involving SnRK2 may have some degree of functional expansion in rice, providing new perspectives for understanding stress signaling complexity in rice.

Beyond phylogenetic classification, our sequence analysis revealed that OsSAPKs share highly conserved kinase domains while exhibiting considerable variation at the C-terminal regulatory segments of these proteins (Figure 2). The conserved catalytic regions among SnRK2 members suggest that the fundamental kinase activity of this family is evolutionarily maintained, whereas differences in regulatory regions and protein interaction patterns may contribute to differences in substrate recognition, protein interactions, or stress-responsive regulation [38]. In particular, the ability of different OsSAPK members from separate phylogenetic groups to associate with OsABI5 raises the possibility that subgroup classification alone may not fully predict protein interaction specificity in rice. Similar evolutionary patterns have been observed in other plant kinase families, where conserved catalytic domains are accompanied by divergent regulatory regions that may influence protein association patterns and regulatory specificity. Despite these structural considerations, the present study identifies candidate OsABI5-interacting kinases based on physical interaction evidence and expression responses under salt-related treatments, rather than establishing their downstream functional consequences. Whether these interactions translate into functional regulation of OsABI5 activity remains unresolved, particularly for Group I and Group II OsSAPKs.

The molecular mechanisms underlying the identified OsSAPK–OsABI5 associations remain unclear. Research in A. thaliana has demonstrated that the conserved ABA signaling cascade involves Group III SnRK2 kinases, which function as upstream regulators of ABA-responsive bZIP transcription factors such as ABI5 and ABFs, through phosphorylation-dependent mechanisms, thereby modulating their activity during ABA responses [38]. Whether the OsSAPK–OsABI5 interactions identified in this study operate through comparable phosphorylation-dependent regulation remains for future investigation. Although OsSAPK9 belongs to the Group III subgroup and may potentially share similarities with the conserved SnRK2–ABI5 regulatory module, its ability to modulate OsABI5 activity requires direct biochemical and genetic validation. The potential functions of Group I and Group II OsSAPKs in association with OsABI5 represent another important area for further exploration. The interaction of OsSAPK1, OsSAPK3, and OsSAPK4 with OsABI5 suggests that these kinases may possess additional regulatory capacities beyond their previously characterized roles. However, the current study does not determine whether these interactions result in ABI5 phosphorylation, changes in ABI5 protein stability, altered DNA-binding activity, or transcriptional regulation of downstream genes. Future biochemical approaches, including in vitro kinase assays, phosphorylation site identification, and functional analyses of phosphorylation-deficient ABI5 variants, will be required to clarify the molecular consequences of these interactions. Furthermore, the distinct evolutionary classification and promoter characteristics of OsSAPKs may provide additional layers of regulation that influence their interaction specificity and stress responsiveness. Comparative analyses among different OsSAPK members may help determine whether specific regulatory regions contribute to their association with OsABI5 or other ABA-related signaling components [39].

Although the present study identifies a set of candidate OsABI5-interacting kinases, further functional characterization is required to determine their biological roles in rice salt stress adaptation. Because an ABA-alone treatment group was not included in the current experimental design, the present study cannot completely distinguish ABA-specific effects from responses caused by the integrated ABA pretreatment and subsequent NaCl exposure. Therefore, the observed phenotypic and transcriptional changes should be interpreted as responses under the combined treatment condition rather than as exclusive ABA- or salt-dependent effects. The identification of OsSAPK1, OsSAPK3, OsSAPK4, and OsSAPK9 provides potential molecular targets for investigating how SnRK2 family diversification contributes to ABA-associated stress regulation in rice. However, whether individual OsSAPK members positively or negatively regulate salt tolerance, and whether their interaction with OsABI5 is required for stress adaptation, remain important questions for future studies. From an applied perspective, these candidate OsSAPK genes may provide useful genetic resources for future rice stress-resistance improvement after their functions have been validated. For example, genetic analyses using CRISPR/Cas9-mediated knockout, overexpression, or allele-specific modification approaches could determine whether specific OsSAPKs contribute to improved salt tolerance under field-relevant conditions. Subsequently, naturally occurring variations in promising OsSAPK alleles could be explored in rice germplasm collections to evaluate their potential associations with salt tolerance-related traits. Such functional validation would be an essential step before applying these genes in molecular breeding programs. Future studies integrating biochemical, genetic, and physiological approaches will be necessary to bridge the current gap between protein interaction evidence and biological function, thereby determining the precise roles of OsSAPKs in ABA-associated salt stress adaptation.

Overall, this study explored the phylogenetic relationships, promoter features, and expression patterns of OsSAPK family members and identifies candidate OsABI5-associated kinases that expand the current understanding of SnRK2-related regulatory networks in rice. These findings establish a foundation for future investigations aimed at linking molecular interactions with stress-responsive phenotypes and ultimately translating basic knowledge of ABA signaling into strategies for improving rice stress resilience.

## 4. Materials and Methods

### 4.1. Phylogenetic and Motif Analysis of SnRK2 Proteins in Plants

SnRK2 family protein sequences were retrieved from the publicly accessible Phytozome database (https://phytozome-next.jgi.doe.gov/). SnRK2 protein sequences were identified from 12 plant species, including *Ananas comosus*, *Arabidopsis thaliana*, *Dioscorea alata*, *Glycine max*, *Gossypium hirsutum*, *Oryza sativa*, *Panicum virgatum*, *Physcomitrium patens*, *Solanum lycopersicum*, *Triticum aestivum*, *Typha latifolia*, and *Zea mays*. Homologous SnRK2 proteins from other species were identified through BLASTP analysis using rice SnRK2 sequences as the input dataset via the Phytozome BLASTP tool (v14, https://phytozome-next.jgi.doe.gov/blast-search (accessed on 12 March 2026)) to identify homologous sequences. The accession information and BLASTP results of the identified SnRK2 homologs are summarized in Table S1. Phylogenetic analysis was performed using MEGA12 software (version 12.0.11, https://www.megasoftware.net/) with the neighbor-joining method and 1000 bootstrap replicates. Subsequently, tree diagram visualization and annotation were realized through the Interactive Tree of Life (https://itol.embl.de/). MEME suite software (version 5.5.9, https://meme-suite.org/meme/tools/meme (accessed on 12 March 2026)) was used for motif analysis of the amino acid C-terminal sequences setting, with 10 motifs being found and zero or one occurrence per sequence (zoops) for site distribution [40].

### 4.2. Promoters Analysis

To analyze cis-regulatory elements, upstream sequences of *OsSAPK* genes were extracted based on the *Oryza sativa* Japonica Group reference genome assembly ASM3414082v1 (GCF_034140825.1) and NCBI gene annotation (RS_2024_06). Gene accession information and corresponding sequence sources are summarized in Table S4. For each *OsSAPK* member, a 2000 bp upstream sequence relative to the translation initiation site (ATG) was extracted from corresponding RefSeq nucleotide records and exported in FASTA format. These sequences were subsequently analyzed using PlantCARE software (https://bioinformatics.psb.ugent.be/webtools/plantcare/html/ (accessed on 10 March 2026)) to analyze the distribution of cis-regulatory motifs in the promoter [41]. The complete list and positional information of predicted cis-elements are provided in Table S2. The identified regulatory elements were further visualized using R software (version 4.6.1) with ggplot2 (version 4.0.3) based on gene functional annotation obtained from eggNOG-mapper GO analysis [42].

### 4.3. Plant Growth, Stress Treatments, and Phenotypic Assays

*Oryza sativa* L. *japonica* cv. Nipponbare (NIP) was used for the expression profiling of *OsSAPK* genes in the study. Rice seeds were germinated with ddH_2_O and then transferred to a hydroponic cultivation system with Yoshida’s rice nutrient solution (Coolaber, Beijing, China, Cat# NSP1040). Seedlings were planted one row apart using 96-well rice hydroponic trays, under a 16 h light (28 ± 2 °C)/8 h dark (25 ± 2 °C) photoperiod in a plant growth chamber [43,44]. Ten-day-old seedlings were pretreated with 1 μM ABA in Yoshida’s rice nutrient solution for 48 h (AS), then subjected to salt stress by transferring them to Yoshida’s nutrient solution supplemented with 100 mM NaCl for 48 h. Seedlings receiving salt treatment alone (S) and untreated seedlings (Ctr) were maintained as independent experimental groups for comparison [45]. Samples from the above-ground part of the seedlings were taken, and the average length and weight of fresh seedlings were measured. The dry matter weight was measured after drying in a 70 °C oven until the weight no longer changed. Five independent biological replicates, each consisting of pooled samples from multiple seedlings, were analyzed for each treatment [46].

### 4.4. Expression Profiling of Rice SAPK Genes

Leaf samples were collected from multiple plants in each group to ensure data reliability. A 200 mg aliquot of frozen rice leaf material was disrupted into a fine powder by adding steel balls to liquid nitrogen. Total RNA extraction was performed by TRIpure Reagent (Aidlab, Beijing, China, Cat# RN01) following the manufacturer’s instructions. First-strand cDNA was reverse-transcribed from 1 μg total RNA using a First-strand cDNA Synthesis Kit (Lablead, Beijing, China, Cat# F0202). The 2x Realab Green PCR Fast mixture (Lablead, Cat# R0202) was used for RT-qPCR in a 20 µL reaction volume [47]. A CFX96 Touch Real-Time PCR Detection System (Bio-Rad, Shanghai, China, Cat# 1855196) was used for RT-qPCR assays, with the reaction conditions listed as follows: 95 °C 30 s, 40 cycles for two steps including 95 °C 10 s and 60 °C 30 s [48,49]. Relative transcript abundance was calculated using the 2^−ΔΔCt^ method, with ubiquitin (LOC_Os03g13170) used as the internal reference gene for normalization. The melting curve analysis was performed after amplification to verify the specificity of PCR products. All primer sequences, melting temperatures (Tm), and amplicon sizes are listed in Table S3, selected via Primer-Blast software (https://www.ncbi.nlm.nih.gov/tools/primer-blast/index.cgi?LINK_LOC=BlastHome (accessed on 14 November 2025)).

### 4.5. Cloning of OsSAPK Genes

DNA fragments encoding the full-length or specific domains of *OsSAPK* genes were commercially synthesized by Sangon Biotech (Shanghai, China). The accession information of the genes used in this study is provided in Table S4. Primers were designed to amplify the corresponding coding sequences from pUC57 templates. All primer sequences, Tm values, and amplicon sizes are provided in Table S3. These fragments were subsequently amplified by PCR using 2xFast Taq plus MasterMix (Labgic, Beijing, China, Cat# BL971B). PCR reactions were carried out with a SimpliAmp™ PCR Thermocycler (Thermo Fisher Scientific, Waltham, MA, USA, A24811) according to the following cycling parameters: 95 °C 30 s, 30 cycles for three steps including 95 °C 10 s, 58 °C 30 s and 72 °C 30 s, and 72 °C, with 5 min for full extension. The amplified PCR products were purified from agarose gels using a Gel Extraction Kit (Tiangen, Beijing, China, DP209) and were ligated into the respective destination vectors, including pGBKT7, pGADT7, pCAMBIA1300-nLUC, pCAMBIA1300-cLUC, pGEX-4T-1, pMAL-c2X, pCAMBIA1300-HA, and pCAMBIA1300-FLAG digested with the restriction enzyme *Bam*HI (Thermo Fisher Scientific, Cat# ER0051) via T4 DNA ligase (Thermo Fisher Scientific, Cat# EL0014) at 16 °C overnight. The vector sequences are provided in Table S5. The ligation products were transformed into *Escherichia coli* DH5α cells (ToloBio, Shanghai, China, Cat# CC96102-2). Positive clones were initially screened by colony PCR and subsequently tested by Sanger sequencing [50].

### 4.6. Yeast Two-Hybrid

Yeast two-hybrid experiments were performed using the Matchmaker™ GAL4 two-hybridization system (Clontech Laboratories, Inc., Mountain View, CA, USA) [51]. The bait (pGBKT7-SAPKs) and prey (pGADT7-ABI5) vectors were co-transformed into a competent *Saccharomyces cerevisiae* AH109 strain (Coolaber, Cat# CC300) [44]. The genotype of the AH109 strain is *MATa*, *trp1-901*, *leu2-3*,*112*, *ura3-52*, *his3-200*, *gal4Δ*, *gal80Δ*, *LYS2::GAL1_UAS_-GAL1_TATA_-HIS3*, *MEL1 GAL2_UAS_-GAL2_TATA_ADE2*, *URA3::MEL1_UAS_-MEL1_TATA_-lacZ*. Co-transformed yeast cells were initially screened on synthetic deletion medium lacking leucine and tryptophan. Subsequently, positive transformants were screened for protein–protein interactions on a quadruple deletion medium lacking adenine, histidine, leucine, and tryptophan. The yeast culture was diluted tenfold in a tenfold gradient and spotted on a selective agar plate. Yeast cells co-transformed with the corresponding empty vectors (pGBKT7 and pGADT7) were used as negative controls.

### 4.7. Luciferase Complementation Imaging Assay

*N. benthamiana* were grown in a mixture (3:1) of peat soil and vermiculite (Macklin, Shanghai, China, Cat# V885843) under a 16 h light/8 h dark photoperiod (22 ± 2 °C) for approximately one month before infiltration [52,53].

*Agrobacterium* GV3101 (Sangon Biotech, Cat# B528430) carrying recombinant vector (SAPKs-pCAMBIA1300-nLUC/ABI5-pCAMBIA1300-cLUC) cultures were harvested using an infiltration buffer containing 10 mM MgCl_2_, 10 mM 2-(N-morpholino)ethanesulfonic acid (MES, pH 5.6), and 150 µM acetosyringone, to adjust to an OD_600_ = 0.6 in the buffer [54]. Leaves of four-week-old *N. benthamiana* infiltrated by mixed bacterial suspensions containing the nLUC and cLUC constructs for 72 h were visualized by spraying with 1 mM D-Luciferin potassium salt solution (Beyotime, Shanghai, China, Cat# ST196) followed by incubation in darkness to imaging [52]. The fluorescence results were detected by imaging system detection using a Tanon 5200 series fully automated chemiluminescence image analysis system (Tanon, Shanghai, China, 5200).

### 4.8. Recombinant Protein Expression and Purification

The recombinant plasmids (*SAPKs*-pGEX-4T-1/*ABI5*-pMAL-c2x) were transformed into *Escherichia coli* BL21 (DE3) (ToloBio, Cat# CC96107-2) for expression induced by 0.5 mM isopropyl-β-D-thiogalactopyranoside (IPTG) (Labgic, Cat# BS119), with a 16 °C shaking incubator overnight. Bacterial cells were collected and lysed by high-pressure homogenization (Antuos Nanotechnology, Suzhou, China, AH-BASIC 30), with a pressure of 800 bar. GST-tag (glutathione S-transferase, Lablead, Cat# P1051) or MBP-tag (maltose-binding protein, Lablead, Cat# M1051) protein purification kits were used, respectively, to isolate the recombination proteins according to the steps in the operator’s manual. Purified proteins were aliquoted and stored at −80 °C.

### 4.9. Pull-Down Assay

Equal molar amounts of purified MBP-OsABI5/MBP protein were co-incubated with GST-OsSAPK protein at 4 °C for 2 h with gentle shaking for in vitro pull-down assay [55]. MBP-tag affinity resin (Lablead, Cat# M1050) was added to the mixture and treated for 30 min at 4 °C. The resin bound to the fusion protein was washed five times with pre-chilled PBS buffer, each time for 5 min on a 4 °C shaker. The binding results were detected by Western blotting and imaged with a Tanon 5200 series fully automated chemiluminescence image analysis system (Tanon, Shanghai, China, 5200).

### 4.10. In Vivo Co-Immunoprecipitation Assay

*Agrobacterium* GV3101 which had been transformed with the recombinant vectors (SAPKs-pCAMBIA1300-HA/ABI5-pCAMBIA1300-FLAG) were adjusted to an OD_600_ = 0.6 in infiltration buffer and injected into the leaves of four-week-old *N. benthamiana* [34]. Infected leaves collected and rapidly frozen by liquid nitrogen after 48 h cultivation were homogenized to a fine powder [35]. Protein extraction used an extracting buffer containing 50 mM Tris-HCl (pH 8.0), 150 mM NaCl, 0.5% NP-40 (Coolaber, Cat# SL9320), 0.2% 2-mercaptoethanol, and 1% protease inhibitor cocktail (Lablead, Cat# C0101) for 1 h on a 4 °C shaker to obtain total protein extracts. The HA-tagged recombinant proteins were immunoprecipitated from the leaf extract total proteins, isolated by adding HA-Nanoab Magnetic Beads (Lablead, Cat# HNM-25-1000) according to the manufacturer’s instructions, and the mixture was incubated at 4 °C in a shaking incubator for 1 h. The samples were washed three times with PBS buffer in a 4 °C shaking incubator for 5 min, and co-immunoprecipitated FLAG-tagged OsABI5 was detected by Western blot using anti-FLAG antibody. Input samples were analyzed in parallel to confirm protein expression.

### 4.11. Statistical Analysis

GraphPad Prism (version 10.1.2, GraphPad Software) was employed for statistical analysis of the experimental data. Data are presented as mean ± standard deviation (SD) from at least three independent biological replicates. Statistical differences among multiple treatment groups were determined using one-way analysis of variance (ANOVA), followed by Tukey’s multiple comparison test. A significance threshold of *p* < 0.05 was used. For figures containing multiple treatment comparisons, statistically significant differences were indicated by different lowercase letters above the corresponding columns.

## 5. Conclusions

This study systematically defines a more diversified SnRK2-ABI5 interactome in rice by integrating transcriptomics, protein interactomics, and in vitro and in vivo biochemical validation. The identification of OsSAPK1, OsSAPK3, OsSAPK4, and OsSAPK9 as direct interacting candidates of OsABI5, including members from Group I and Group II SnRK2 subgroups, expands the current understanding of the potential diversity of SnRK2-associated regulatory relationships in rice ABA signaling. These findings suggest that OsABI5-associated kinase interactions may extend beyond the classical Group III SnRK2-mediated model described in *A. thaliana*, providing new perspectives for investigating the diversification of ABA-related stress signaling networks in monocot crops. However, the physiological functions and downstream regulatory consequences of these interactions remain to be experimentally validated. Future studies combining genetic approaches, phosphorylation analyses, and transcriptomic investigations will be required to determine how individual OsSAPK–OsABI5 modules contribute to salt stress adaptation. Following functional validation, these candidate genes may provide valuable resources for improving stress resilience in rice through molecular breeding strategies.

## Data Availability

The data presented in this study are available in the Phytozome database at https://phytozome-next.jgi.doe.gov/. Reference number [56].

## Supplementary Materials

The following supporting information can be downloaded at: https://www.mdpi.com/article/10.3390/ijms27156788/s1.

## Abbreviations

ABA	Abscisic acid
PP2C	Protein phosphatase 2C
SnRK2	Sucrose Non-Fermenting 1-Related Protein Kinase 2
SAPKs	Stress-activated protein kinases
ABRE	ABA response elements
ABF	ABA response element-binding factors
ABI5	ABA insensitive factor 5
MBP	Maltose-binding protein
GST	Glutathione-S-transferase
Leu	Leucine
Trp	Tryptophan
Ade	Adenine
His	Histidine
PCR	Polymerase chain reaction
RT-qPCR	Quantitative real-time polymerase chain reaction
