# Supplementary material for: Evolutionary Characteristics of the Rice SnRK2 Family and ABI5 Interaction Analysis Reveal Their Key Regulatory Role in Osmotic Stress Signaling
Source: Int J Mol Sci. 2026 Jul 29;27(15):6788. doi: 10.3390/ijms27156788 (PMC13467227; doi:10.3390/ijms27156788)
Supplement: Supplementary file 1 [file ijms-27-06788-s001.zip › Figure S1. Multiple Sequence Alignment of Plant SnRK2 Amino Acids..pdf]

A

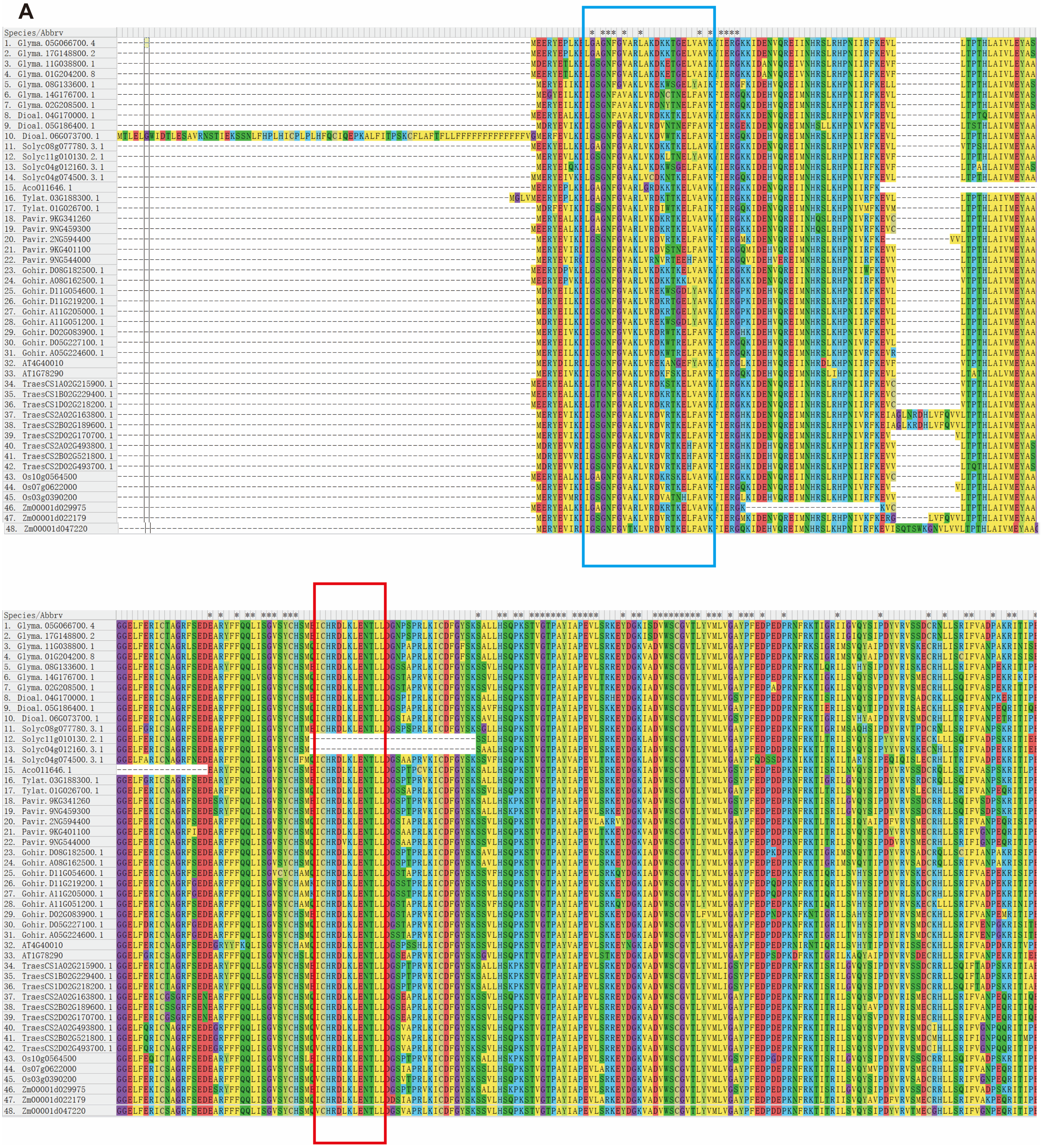

Species/Abbrv
1. Glyma.05G066700.4
2. Glyma.17G148800.2
3. Glyma.11G038800.1
4. Glyma.01G204200.8
5. Glyma.08G133600.1
6. Glyma.14G176700.1
7. Glyma.02G208500.1
8. Dioal.04G170000.1
9. Dioal.05G186400.1
10. Dioal.06G073700.1
11. Solyc08g077780.3.1
12. Solyc11g010130.2.1
13. Solyc04g012160.3.1
14. Solyc04g074500.3.1
15. Aco011646.1
16. Tylat.03G188300.1
17. Tylat.01G026700.1
18. Pavir.9KG341260
19. Pavir.9NG459300
20. Pavir.2NG594400
21. Pavir.9KG401100
22. Pavir.9NG544000
23. Gohir.D08G182500.1
24. Gohir.A08G162500.1
25. Gohir.D11G054600.1
26. Gohir.D11G219200.1
27. Gohir.A11G205000.1
28. Gohir.A11G051200.1
29. Gohir.D02G083900.1
30. Gohir.D05G227100.1
31. Gohir.A05G224600.1
32. AT4G40010
33. AT1G78290
34. TraesCS1A02G215900.1
35. TraesCS1B02G229400.1
36. TraesCS1D02G218200.1
37. TraesCS2A02G163800.1
38. TraesCS2B02G189600.1
39. TraesCS2D02G170700.1
40. TraesCS2A02G493800.1
41. TraesCS2B02G521800.1
42. TraesCS2D02G493700.1
43. Os10g0564500
44. Os07g0622000
45. Os03g0390200
46. Zm00001d029975
47. Zm00001d022179
48. Zm00001d047220

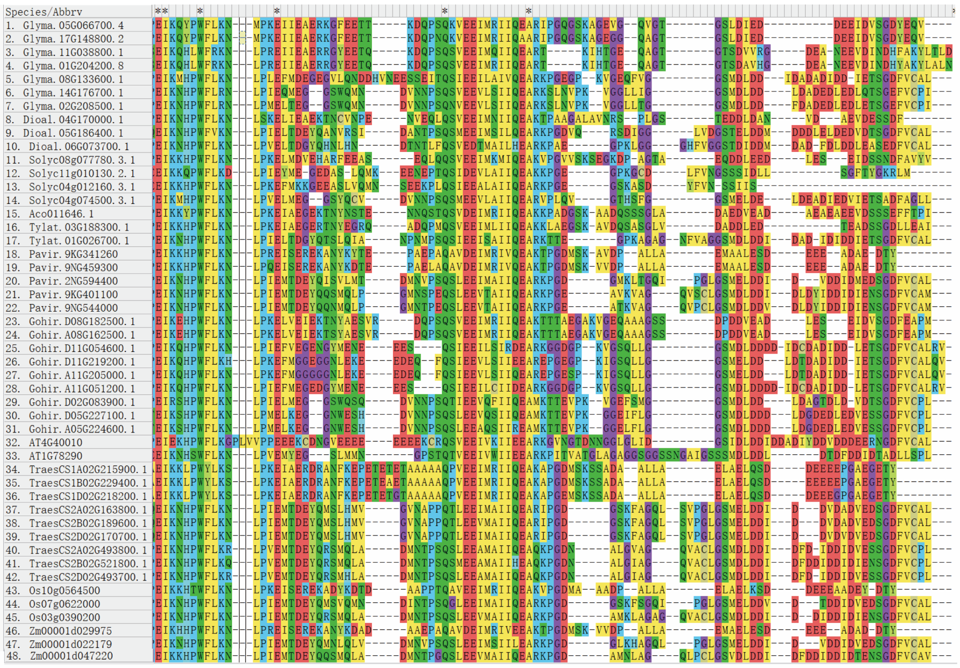
Species/Abbrv
1. Glyma.05G066700.4
2. Glyma.17G148800.2
3. Glyma.11G038800.1
4. Glyma.01G204200.8
5. Glyma.08G133600.1
6. Glyma.14G176700.1
7. Glyma.02G208500.1
8. Dioal.04G170000.1
9. Dioal.05G186400.1
10. Dioal.06G073700.1
11. Solyc08g077780.3.1
12. Solyc11g010130.2.1
13. Solyc04g012160.3.1
14. Solyc04g074500.3.1
15. Aco011646.1
16. Tylat.03G188300.1
17. Tylat.01G026700.1
18. Pavir.9KG341260
19. Pavir.9NG459300
20. Pavir.2NG594400
21. Pavir.9KG401100
22. Pavir.9NG544000
23. Gohir.D08G182500.1
24. Gohir.A08G162500.1
25. Gohir.D11G054600.1
26. Gohir.D11G219200.1
27. Gohir.A11G205000.1
28. Gohir.A11G051200.1
29. Gohir.D02G083900.1
30. Gohir.D05G227100.1
31. Gohir.A05G224600.1
32. AT4G40010
33. AT1G78290
34. TraesCS1A02G215900.1
35. TraesCS1B02G229400.1
36. TraesCS1D02G218200.1
37. TraesCS2A02G163800.1
38. TraesCS2B02G189600.1
39. TraesCS2D02G170700.1
40. TraesCS2A02G493800.1
41. TraesCS2B02G521800.1
42. TraesCS2D02G493700.1
43. Os10g0564500
44. Os07g0622000
45. Os03g0390200
46. Zm00001d029975
47. Zm00001d022179
48. Zm00001d047220

B

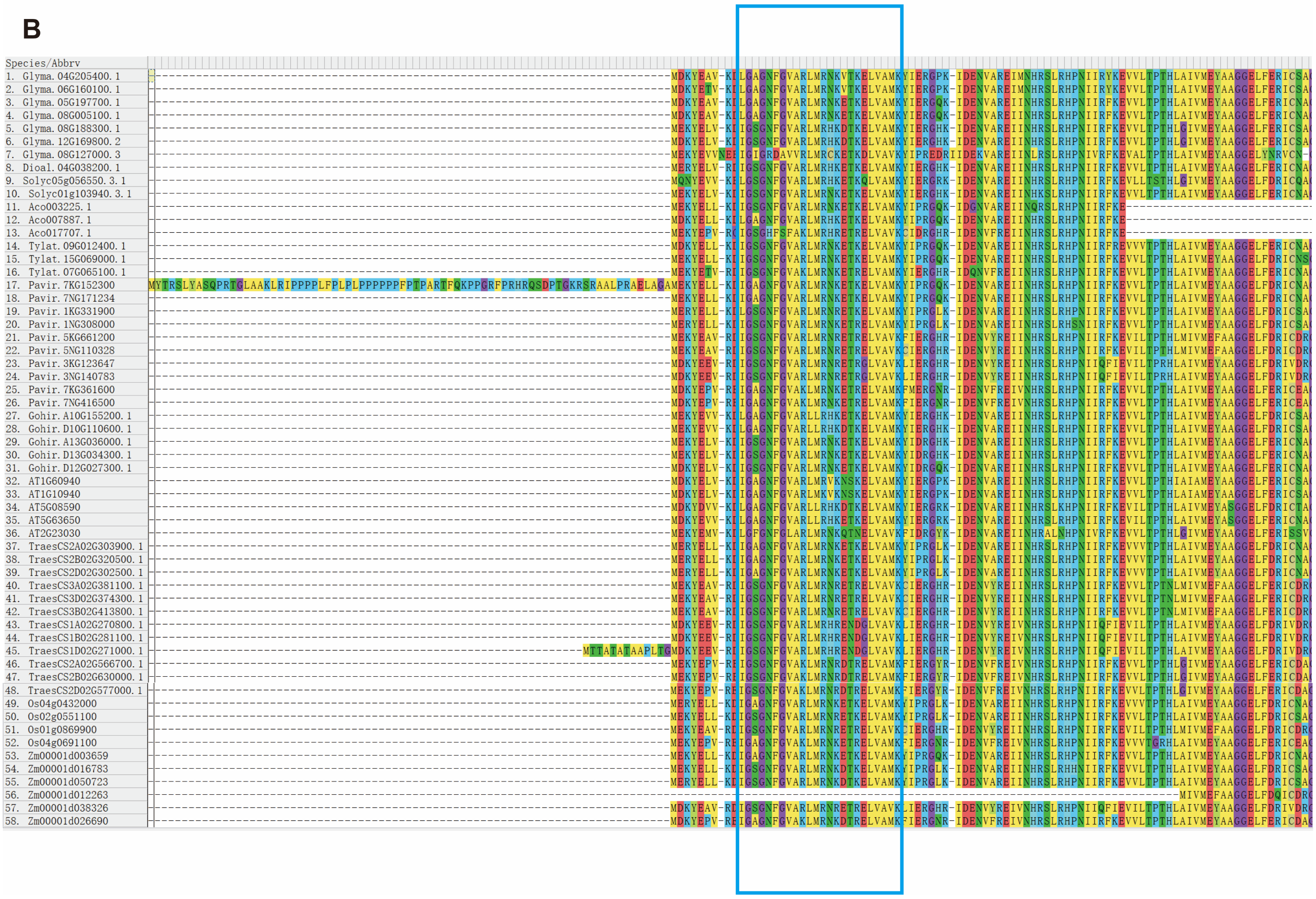
Species/Abbrv
1. Glyma.04G205400.1
2. Glyma.06G160100.1
3. Glyma.05G197700.1
4. Glyma.08G005100.1
5. Glyma.08G188300.1
6. Glyma.12G169800.2
7. Glyma.08G127000.3
8. Dioal.04G038200.1
9. Solyc05g056550.3.1
10. Solyc01g103940.3.1
11. Aco003225.1
12. Aco007887.1
13. Aco017707.1
14. Tylat.09G012400.1
15. Tylat.15G069000.1
16. Tylat.07G065100.1
17. Pavir.7KG152300
18. Pavir.7NG171234
19. Pavir.1KG331900
20. Pavir.1NG308000
21. Pavir.5KG661200
22. Pavir.5NG110328
23. Pavir.3KG123647
24. Pavir.3NG140783
25. Pavir.7KG361600
26. Pavir.7NG416500
27. Gohir.A10G155200.1
28. Gohir.D10G110600.1
29. Gohir.A13G036000.1
30. Gohir.D13G034300.1
31. Gohir.D12G027300.1
32. AT1G60940
33. AT1G10940
34. AT5G08590
35. AT5G63650
36. AT2G23030
37. TraesCS2A02G303900.1
38. TraesCS2B02G320500.1
39. TraesCS2D02G302500.1
40. TraesCS3A02G381100.1
41. TraesCS3D02G374300.1
42. TraesCS3B02G413800.1
43. TraesCS1A02G270800.1
44. TraesCS1B02G281100.1
45. TraesCS1D02G271000.1
46. TraesCS2A02G566700.1
47. TraesCS2B02G630000.1
48. TraesCS2D02G577000.1
49. Os04g0432000
50. Os02g0551100
51. Os01g0869900
52. Os04g0691100
53. Zm00001d003659
54. Zm00001d016783
55. Zm00001d050723
56. Zm00001d012263
57. Zm00001d038326
58. Zm00001d026690

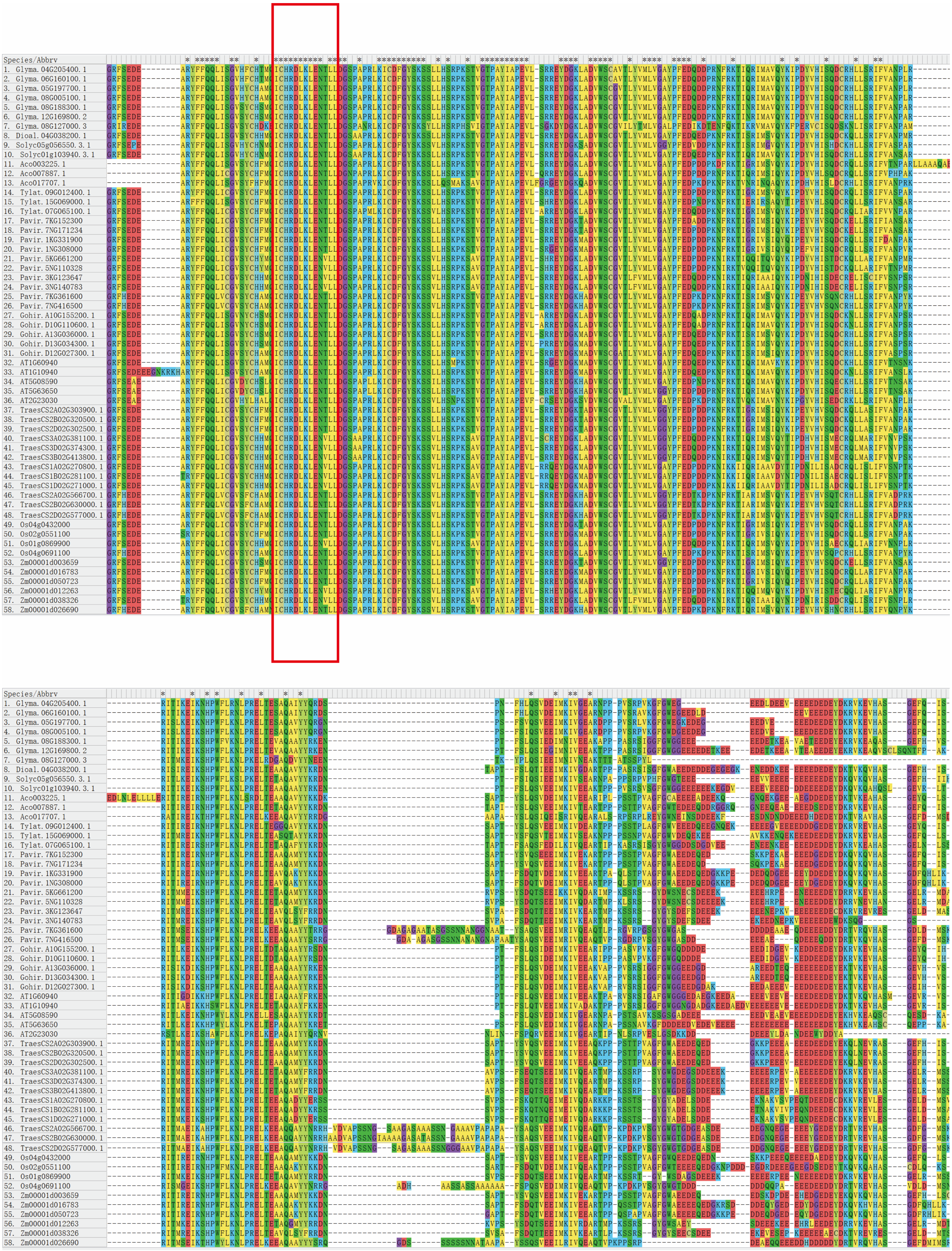
Species/Abbrv
1. Glyma.04G205400.1
2. Glyma.06G160100.1
3. Glyma.05G197700.1
4. Glyma.08G005100.1
5. Glyma.08G188300.1
6. Glyma.12G169800.2
7. Glyma.08G127000.3
8. Dioal.04G038200.1
9. Solyc05g056550.3.1
10. Solyc01g103940.3.1
11. Aco003225.1
12. Aco007887.1
13. Aco017707.1
14. Tylat.09G012400.1
15. Tylat.15G069000.1
16. Tylat.07G065100.1
17. Pavir.7KG152300
18. Pavir.7NG171234
19. Pavir.1KG331900
20. Pavir.1NG308000
21. Pavir.5KG661200
22. Pavir.5NG110328
23. Pavir.3KG123647
24. Pavir.3NG140783
25. Pavir.7KG361600
26. Pavir.7NG416500
27. Gohir.A10G155200.1
28. Gohir.D10G110600.1
29. Gohir.A13G036000.1
30. Gohir.D13G034300.1
31. Gohir.D12G027300.1
32. AT1G60940
33. AT1G10940
34. AT5G08590
35. AT5G63650
36. AT2G23030
37. TraesCS2A02G303900.1
38. TraesCS2B02G320500.1
39. TraesCS2D02G302500.1
40. TraesCS3A02G381100.1
41. TraesCS3D02G374300.1
42. TraesCS3B02G413800.1
43. TraesCS1A02G270800.1
44. TraesCS1B02G281100.1
45. TraesCS1D02G271000.1
46. TraesCS2A02G566700.1
47. TraesCS2B02G630000.1
48. TraesCS2D02G577000.1
49. Os04g0432000
50. Os02g0551100
51. Os01g0869900
52. Os04g0691100
53. Zm00001d003659
54. Zm00001d016783
55. Zm00001d050723
56. Zm00001d012263
57. Zm00001d038326
58. Zm00001d026690

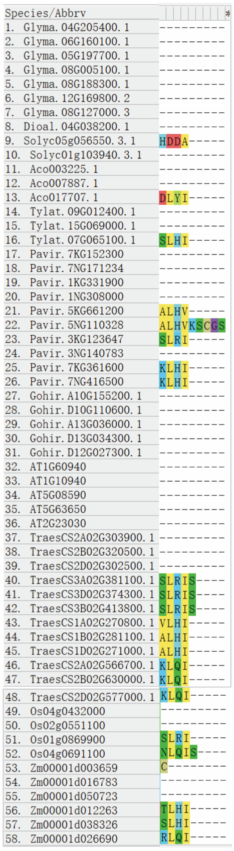
Species/Abbrv
1. Glyma.04G205400.1
2. Glyma.06G160100.1
3. Glyma.05G197700.1
4. Glyma.08G005100.1
5. Glyma.08G188300.1
6. Glyma.12G169800.2
7. Glyma.08G127000.3
8. Dioal.04G038200.1
9. Solyc05g056550.3.1
10. Solyc01g103940.3.1
11. Aco003225.1
12. Aco007887.1
13. Aco017707.1
14. Tylat.09G012400.1
15. Tylat.15G069000.1
16. Tylat.07G065100.1
17. Pavir.7KG152300
18. Pavir.7NG171234
19. Pavir.1KG331900
20. Pavir.1NG308000
21. Pavir.5KG661200
22. Pavir.5NG110328
23. Pavir.3KG123647
24. Pavir.3NG140783
25. Pavir.7KG361600
26. Pavir.7NG416500
27. Gohir.A10G155200.1
28. Gohir.D10G110600.1
29. Gohir.A13G036000.1
30. Gohir.D13G034300.1
31. Gohir.D12G027300.1
32. AT1G60940
33. AT1G10940
34. AT5G08590
35. AT5G63650
36. AT2G23030
37. TraesCS2A02G303900.1
38. TraesCS2B02G320500.1
39. TraesCS2D02G302500.1
40. TraesCS3A02G381100.1
41. TraesCS3D02G374300.1
42. TraesCS3B02G413800.1
43. TraesCS1A02G270800.1
44. TraesCS1B02G281100.1
45. TraesCS1D02G271000.1
46. TraesCS2A02G566700.1
47. TraesCS2B02G630000.1
48. TraesCS2D02G577000.1
49. Os04g0432000
50. Os02g0551100
51. Os01g0869900
52. Os04g0691100
53. Zm00001d003659
54. Zm00001d016783
55. Zm00001d050723
56. Zm00001d012263
57. Zm00001d038326
58. Zm00001d026690

C

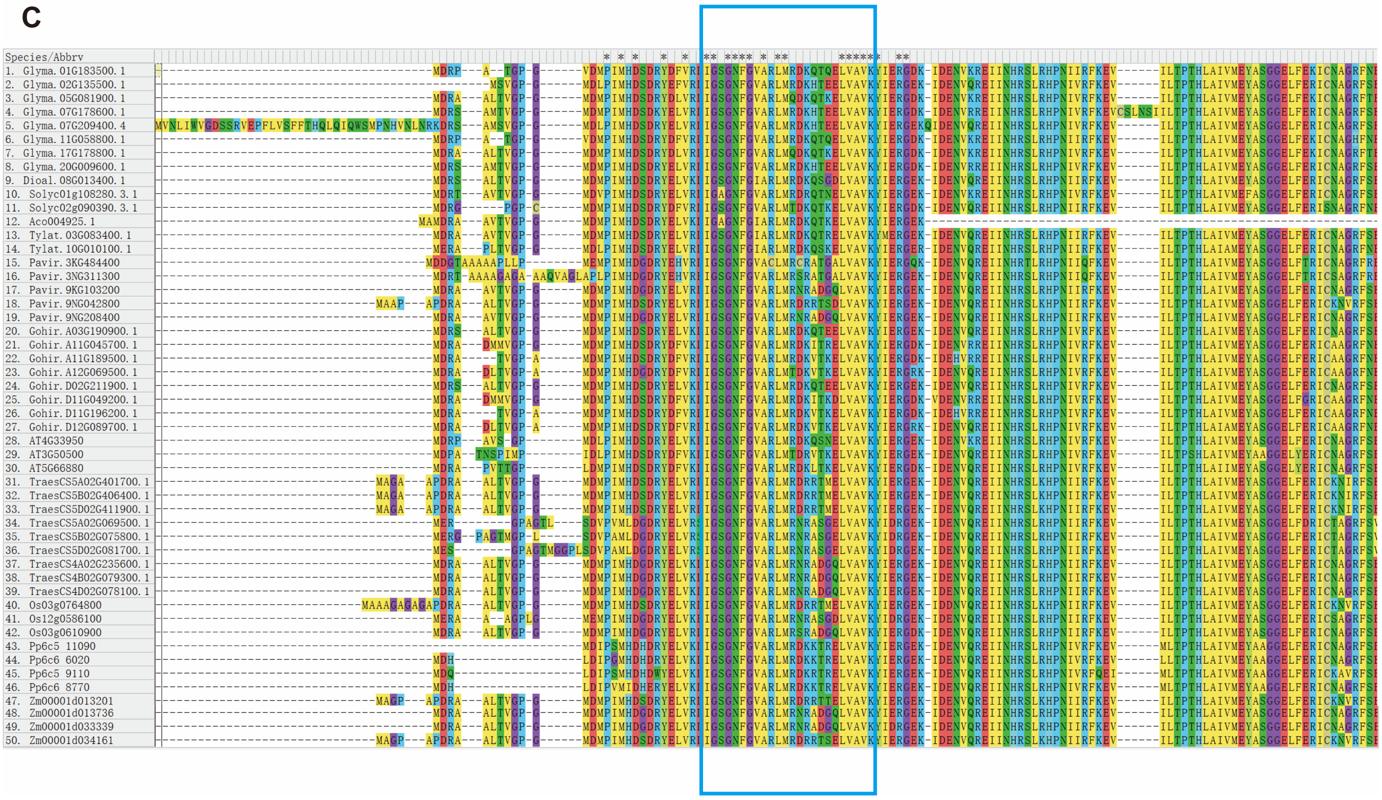
Species/Abbrv
1. Glyma.01G183500.1
2. Glyma.02G135500.1
3. Glyma.05G081900.1
4. Glyma.07G178600.1
5. Glyma.07G209400.4
6. Glyma.11G058800.1
7. Glyma.17G178800.1
8. Glyma.20G009600.1
9. Dioal.08G013400.1
10. Solyc01g108280.3.1
11. Solyc02g090390.3.1
12. Aco004925.1
13. Tylat.03G083400.1
14. Tylat.10G010100.1
15. Pavir.3KG484400
16. Pavir.3NG311300
17. Pavir.9KG103200
18. Pavir.9NG042800
19. Pavir.9NG208400
20. Gohir.A03G190900.1
21. Gohir.A11G045700.1
22. Gohir.A11G189500.1
23. Gohir.A12G069500.1
24. Gohir.D02G211900.1
25. Gohir.D11G049200.1
26. Gohir.D11G196200.1
27. Gohir.D12G089700.1
28. AT4G33950
29. AT3G50500
30. AT5G66880
31. TraesCS5A02G401700.1
32. TraesCS5B02G406400.1
33. TraesCS5D02G411900.1
34. TraesCS5A02G069500.1
35. TraesCS5B02G075800.1
36. TraesCS5D02G081700.1
37. TraesCS4A02G235600.1
38. TraesCS4B02G079300.1
39. TraesCS4D02G078100.1
40. Os03g0764800
41. Os12g0586100
42. Os03g0610900
43. Pp6c5_11090
44. Pp6c6_6020
45. Pp6c5_9110
46. Pp6c6_8770
47. Zm00001d013201
48. Zm00001d013736
49. Zm00001d033339
50. Zm00001d034161

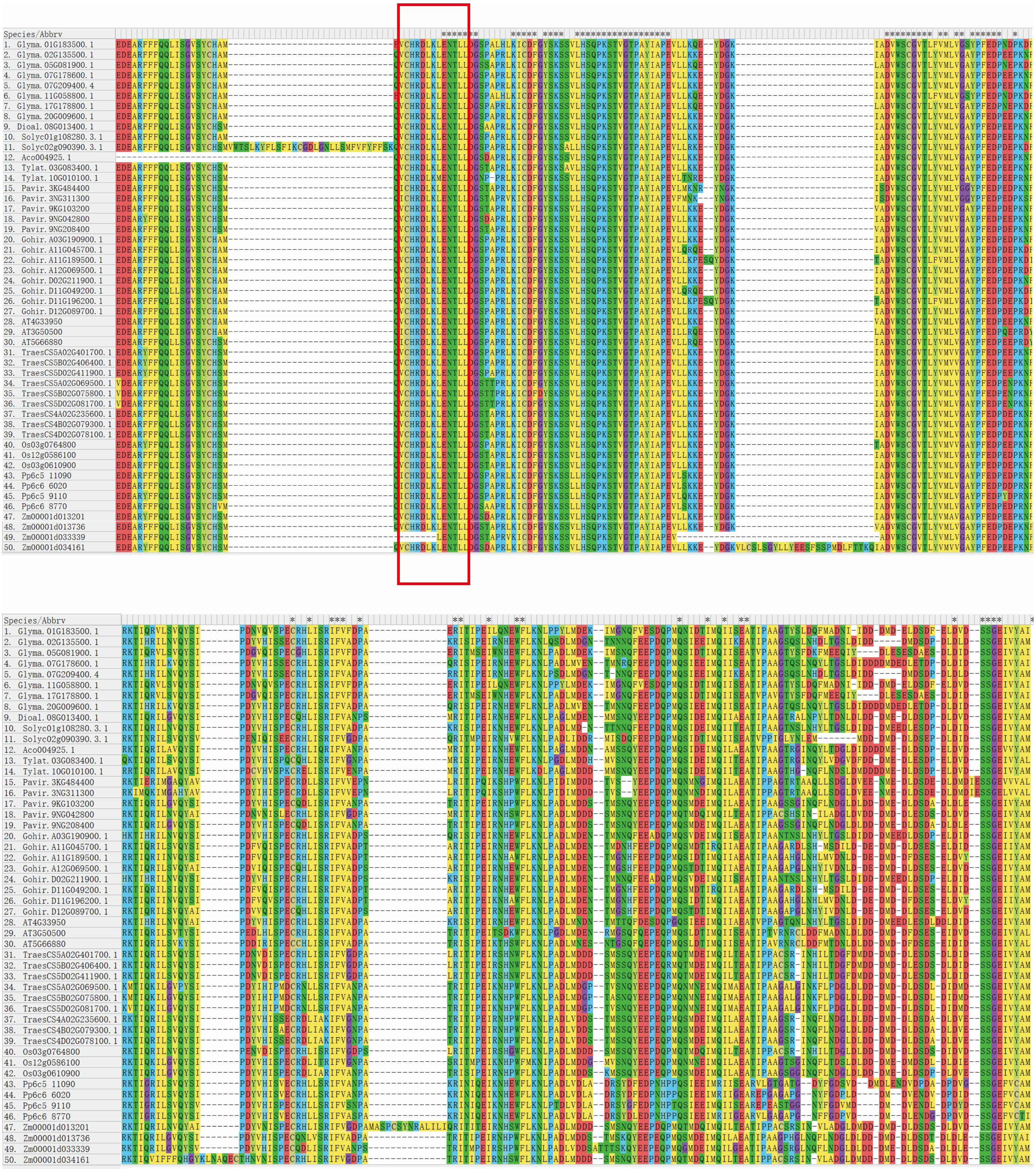

Figure S1. Multiple Sequence Alignment of Plant SnRK2 Amino Acids. Ser/Thr protein kinase activity sites and ATP-connecting sites were identified in all plant SnRK2 members from group I(A)/group II(B)/group III(C) respectively by Red and Green boxes in this study. Different colored patches are used to label different amino acids. Asterisks (*) indicate amino acid positions that are completely conserved among all aligned SnRK2 members.
