# Supplementary material for: Evolutionary Characteristics of the Rice SnRK2 Family and ABI5 Interaction Analysis Reveal Their Key Regulatory Role in Osmotic Stress Signaling
Source: Int J Mol Sci. 2026 Jul 29;27(15):6788. doi: 10.3390/ijms27156788 (PMC13467227; doi:10.3390/ijms27156788)

Figure 7. A

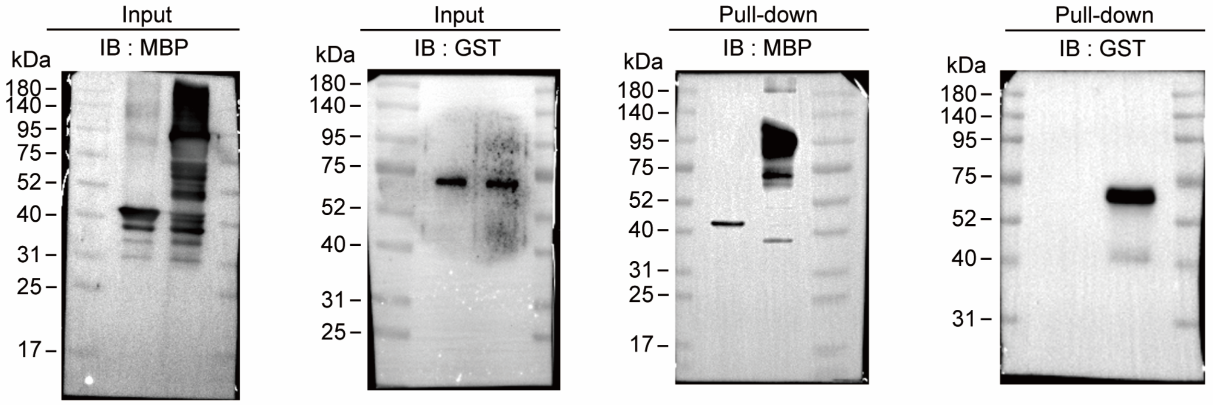

Figure 7. B

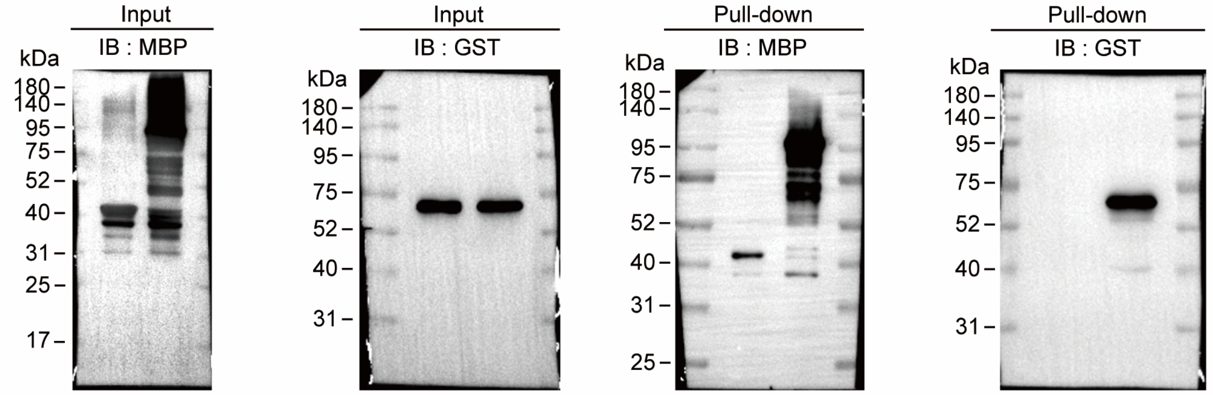

Figure 7. C

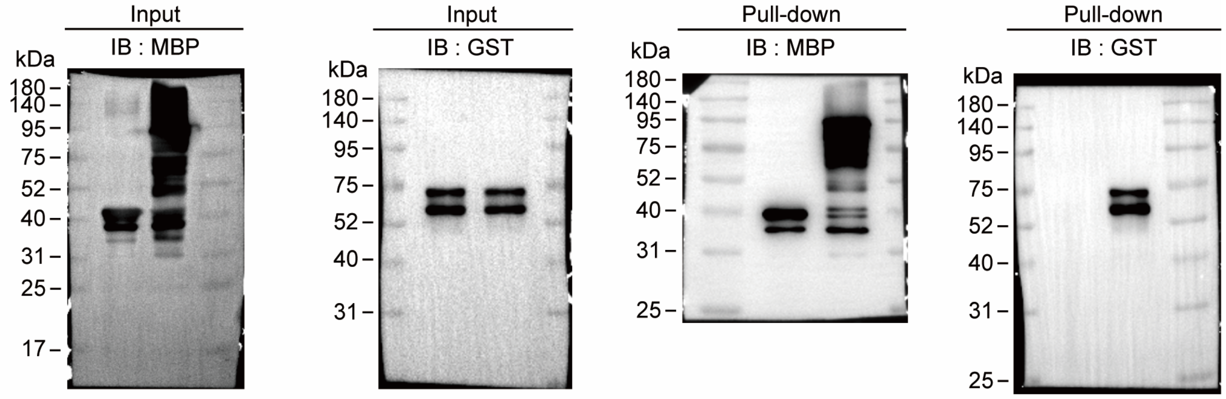

Figure 7. D

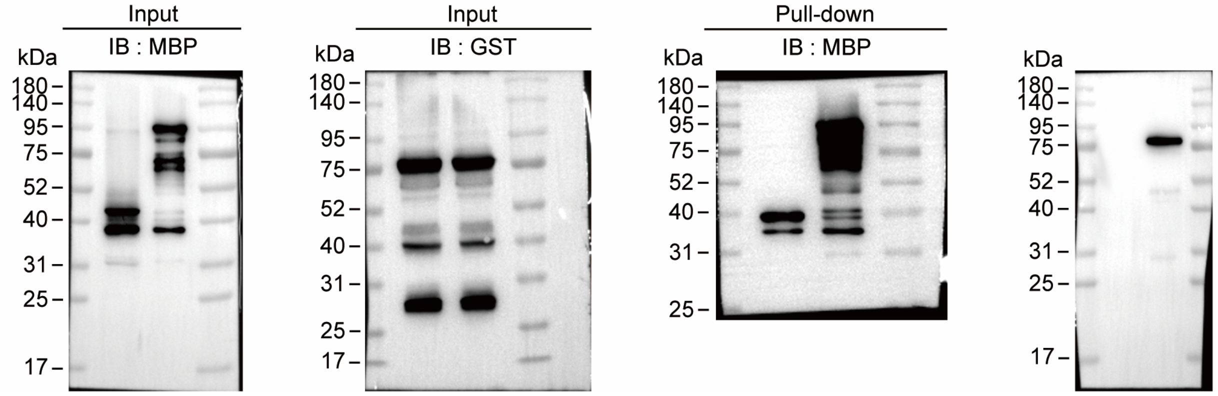

Figure 7. E

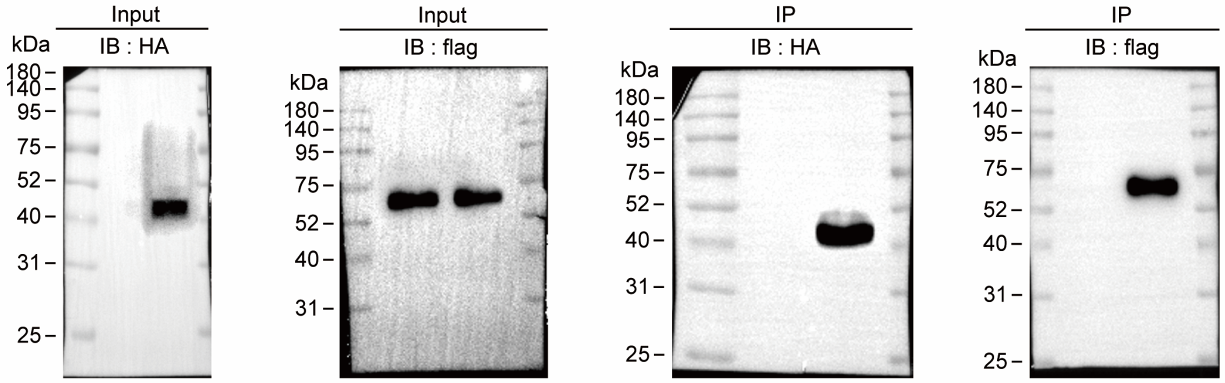

Figure 7. F

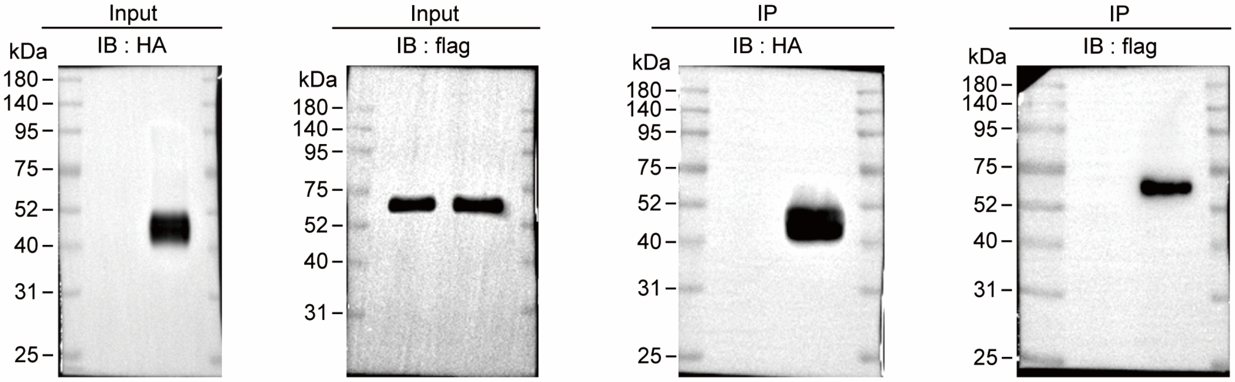

Figure 7. G

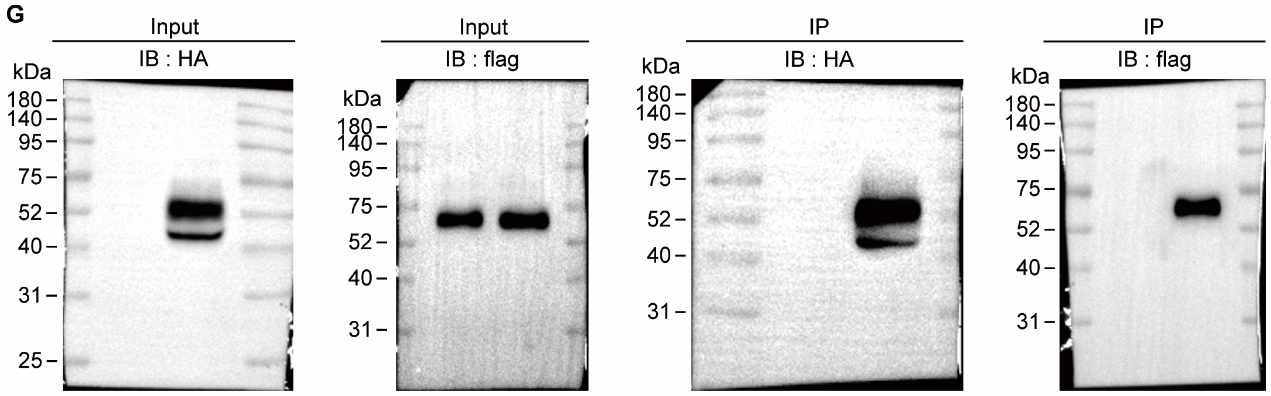

Figure 7. H

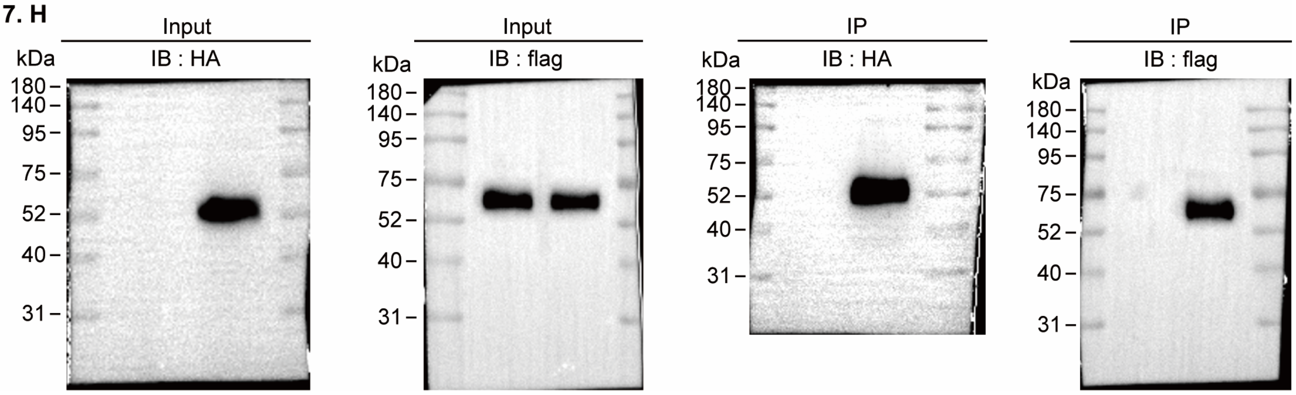

Supplement: Supplementary file 1 [file ijms-27-06788-s001.zip › Figure S2 Original Image.pdf]
